# Precursors of Majorana modes and their length-dependent energy oscillations probed at both ends of atomic Shiba chains

**DOI:** 10.1038/s41565-022-01078-4

**Published:** 2022-03-07

**Authors:** Lucas Schneider, Philip Beck, Jannis Neuhaus-Steinmetz, Levente Rózsa, Thore Posske, Jens Wiebe, Roland Wiesendanger

**Affiliations:** 1 Department of Physics, Universität Hamburg, Hamburg, Germany; 2 Department of Physics, University of Konstanz, Konstanz, Germany; 3 I. Institute for Theoretical Physics, Universität Hamburg, Hamburg, Germany; 4 The Hamburg Centre for Ultrafast Imaging, Luruper Chaussee, Hamburg, Germany

**Keywords:** Superconducting properties and materials, Topological defects

## Abstract

Isolated Majorana modes (MMs) are highly non-local quantum states with non-Abelian exchange statistics, which localize at the two ends of finite-size 1D topological superconductors of sufficient length. Experimental evidence for MMs is so far based on the detection of several key signatures: for example, a conductance peak pinned to the Fermi energy or an oscillatory peak splitting in short 1D systems when the MMs overlap. However, most of these key signatures were probed only on one of the ends of the 1D system, and firm evidence for an MM requires the simultaneous detection of all the key signatures on both ends. Here we construct short atomic spin chains on a superconductor—also known as Shiba chains—up to a chain length of 45 atoms using tip-assisted atom manipulation in scanning tunnelling microscopy experiments. We observe zero-energy conductance peaks localized at both ends of the chain that simultaneously split off from the Fermi energy in an oscillatory fashion after altering the chain length. By fitting the parameters of a low-energy model to the data, we find that the peaks are consistent with precursors of MMs that evolve into isolated MMs protected by an estimated topological gap of 50 μeV in chains of at least 35 nm length, corresponding to 70 atoms.

## Abstract (web summary)

Majorana modes are highly non-local quantum states with non-Abelian exchange statistics, which localize at the two ends of finite-size 1D topological superconductors of sufficient length. By precisely positioning magnetic atoms on a superconducting surface, their interaction is tailored such that the precursors of Majorana modes are simultaneously observed on both ends of linear atomic chains.

## Main

Realizing isolated Majorana modes (MMs) as zero-energy excitations in solid-state systems has been an immense quest in the past two decades, being motivated by their possible use for fault-tolerant topological quantum computing^1–3^. Theoretical proposals combine superconductivity, magnetism and Rashba spin–orbit coupling (SOC)^4–10^. One-dimensional (1D) experimental platforms featuring these effects include semiconducting nanowires in proximity to superconductors with an externally applied Zeeman magnetic field^11,12^ or atomic spin chains with ferromagnetic^13–16^ or spin-helical order^17,18^ on superconducting substrates. MMs on the system’s boundaries are the consequence of a topologically non-trivial band structure in the chain’s bulk. This makes them immune to perturbations sufficiently local compared with the size of the system. Atomic spin chains studied so far are short, consisting of only tens of atoms^13,17,18^. Here MMs on both ends of the chain may still interact, thereby splitting in energy away from zero in an oscillatory fashion as a function of the chain length, one of the key signatures of the so-called precursors of MMs (PMMs) in short chains^19–21^. Indeed, Coulomb blockade spectroscopy in InAs nanowires coupled to Al has provided evidence for an oscillatory splitting of near-zero-energy states as a function of the Zeeman field, which decreased for longer devices^20^. However, the length could not be continuously varied in these measurements and they were done for only one of the wires’ ends. Another indication for MMs, the quantized zero-bias conductance, has been detected only on one of the ends of InSb nanowires coupled to NbTiN, whereas the other simultaneously measured end showed a different signature^22^. Zero-bias peaks as indications for MMs or their precursors have also been observed at the ends of atomic spin chains^13–16^. However, such peaks were only found for some of the chains and were not detected on both ends of the same defect-free chain, whereas other chains in the same system did not display this signature at all^13–16^. Also, it was not possible to continuously vary the length of the chains. In this work, we measure the energy oscillations of PMMs in Mn chains on Nb(110), along the entire chain, including both ends, and as a function of the chain length that we continuously vary in an atom-by-atom manner. Using this extensive dataset, we can determine all the parameters of a low-energy model^9,23^, except for an effective Rashba SOC whose order is deduced from first-principles calculations. We predict the chain length above which isolated and topologically protected MMs will evolve from these PMMs.

### Topological phase diagram of Shiba chains

Topological superconductivity and the resulting MMs can be engineered in 1D ferromagnets with an odd number of spin-polarized bands crossing the Fermi energy *E*_F_ (refs. ^4,5,9^). The low-energy bands may be formed by hybridizing the Yu–Shiba–Rusinov (YSR)^9,10,23^ states locally induced by magnetic impurities on superconducting substrates^24–27^. This has led to the intense investigation of YSR states in the past years^24,28–32^. Recently, it has been shown that the dispersions of emergent YSR bands can be measured in Mn spin chains along the [001] direction on Nb(110) (ref. ^23^). Experimental evidence for *p*-wave correlations in this system was found, leading to gap *Δ*_*p*_ exceeding the energy splittings of the states due to finite-size quantization. However, the multiband nature of the YSR chain prevented the identification of its total topological phase. In the following, we exploit this microscopic insight into the low-energy band formation to design an effective one-band system from single, hybridizing YSR states in a bottom-up approach. In this scenario, the system is topologically non-trivial in the presence of any finite *p*-wave pairing, as shown below.

**Fig. 1:**
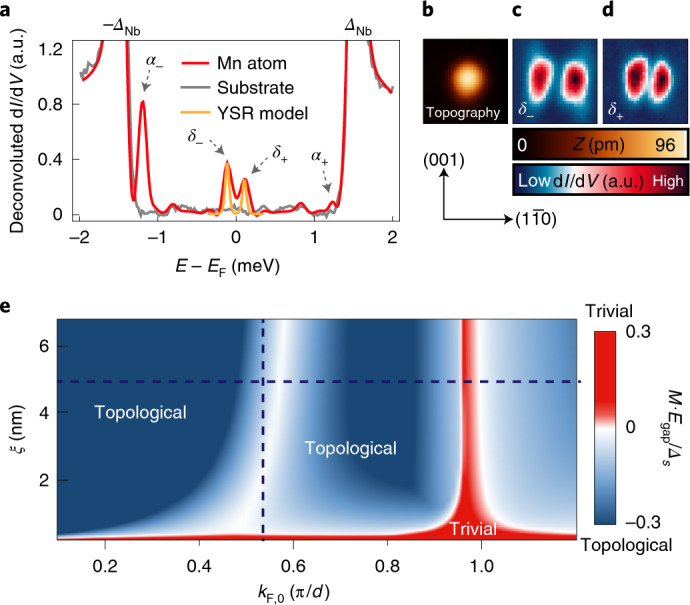
YSR states of single Mn atoms on Nb(110) and resulting topological properties of a hybridized chain. **a**, Deconvoluted d*I*/d*V* spectra averaged around a single Mn atom on Nb(110) and on the bare substrate. Pairs of the most-intense YSR states, namely, *δ*_+/–_ and *α*_+/–_, are highlighted by arrows. The weak resonances at ±0.8 meV are due to additional YSR states, which can be disregarded here (Supplementary Note 1). The measured coherence peaks are energetically located at the superconducting gap of bulk Nb (*Δ*_Nb_ = 1.52 meV). Both energy and particle–hole asymmetry of the *δ*-YSR states are well described by a YSR model with the magnetic and non-magnetic scattering parameters *A* = 1.1 and *B* = 0.2 (orange line; Methods). **b**, STM image of a single Mn atom. **c**,**d**, Spatially resolved d*I*/d*V* map around the atom at energies of *δ*_–_ (**c**) and *δ*_+_ (**d**), showing the state’s lobes along [$1\bar 10$]—the direction of the chains (image sizes are 2 × 2 nm^2^). **e**, Topological phase diagram calculated for an infinite chain of interacting YSR states for different values of Fermi wavevector *k*_F,0_ and coherence length *ξ* of the substrate. The parameters for the YSR states (as derived in **a**) are used; a constant, weak effective Rashba SOC *k*_h_ = 0.05π/*d* is added. The phase diagram does not qualitatively change for other small values of *k*_h_. The colour scale indicates the magnitude of the system’s gap *E*_gap_ multiplied by the topological ${\Bbb Z}_2$ invariant, which is *M* = −1 (topologically non-trivial; blue) or *M* = +1 (trivial; red), compared with the superconducting pairing of the substrate, *Δ*_*s*_. The dark dashed lines mark the parameters used in Fig. 4 that best describe the experimental data in Fig. 3. Source data

**Fig. 2:**
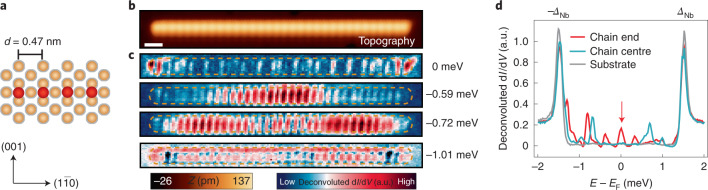
In-gap states in Mn chains on Nb(110) along [$1\bar 10$]. **a**, Geometry of the experimentally assembled Mn atoms (red spheres) on top of the atoms of the superconducting Nb host (brown spheres). **b**, Constant-current STM image (topography) of a Mn_32_ chain. Scale bar, 1 nm. **c**, Corresponding deconvoluted d*I*/d*V* maps at the indicated energies. The brown dashed lines mark the position of the chain. **d**, Single deconvoluted d*I*/d*V* spectra obtained on the chain’s end, in the centre and on the Nb substrate. The zero-energy peak is highlighted by the red arrow. Source data

**Extended Data Fig. 1:**
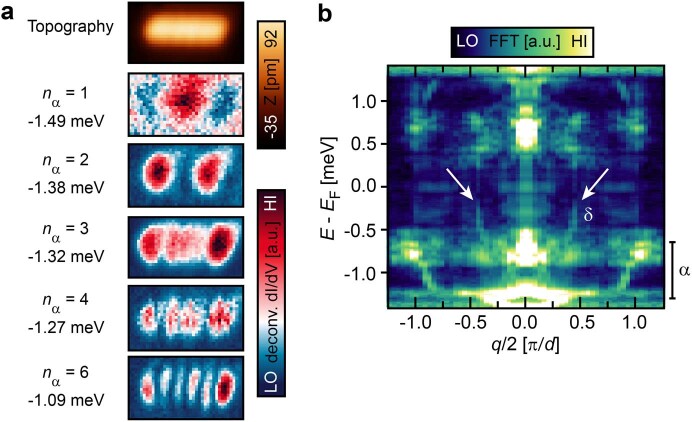
Quasiparticle interference patterns of sub-gap states. **a**, STM image (topography, top panel) and simultaneously recorded d*I*/d*V* maps (bottom panels) of a Mn_6_ chain showing confined states close to the negative energy coherence peak. These can be interpreted as QPI patterns originating from a YSR band of this chain related to one of the multiorbital YSR states, most probably the α-YSR state^23^. This state differs from the *δ*-YSR state that determines the low energy band structure of the chain investigated in the main text. **b**, QPI pattern extracted from Mn chains of length *N* = 10 to *N* = 32 where *q* is the scattering wavevector. The arrows indicate the position of a band crossing $E_{\mathrm{F}}$ at $k_{\mathrm{F}} \approx \pm \pi /2d$ which has the orbital character of the *δ*-YSR state. The range indicated with α on the right edge indicates the width of the band most probably originating from the α-YSR states. Parameters: *V*_stab_ = −6 mV, *I*_stab_ = 1 nA, *V*_mod_ = 20 µV. Source data

**Extended Data Fig. 2:**
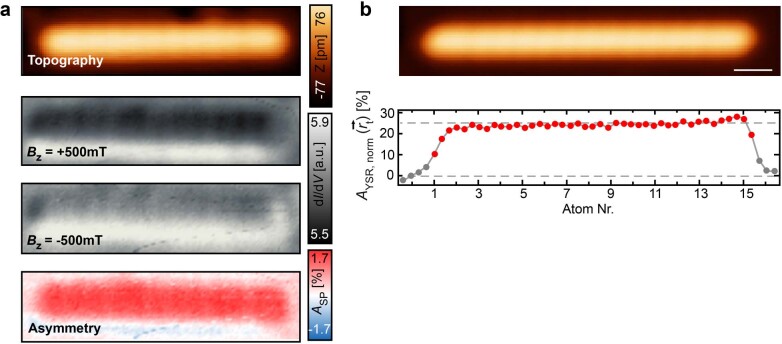
Spin-polarized measurements of the magnetic ground state of Mn chains. **a**, STM image (top panel) and simultaneously recorded d*I*/d*V* maps (two middle panels) of a Mn_11_ chain in an external field of *B*_z_ = ±0.5 T recorded with a bulk Cr tip. Mapping the d*I*/d*V* asymmetry (bottom panel) defined via *A*_SP_ = (d*I*/d*V*|_−0.5T_ - d*I*/d*V*|_+0.5T_)/ (d*I*/d*V*|_−0.5T_ + d*I*/d*V*|_+0.5T_), we find that the contrast on the chain is homogeneous, thereby proving the ferromagnetic alignment of the chain’s magnetic moments. Parameters: *V*_d.c._ = 6 mV, *I* = 1 nA, *V*_mod_ = 2 mV. **b**, STM image (top) and spin-contrast $A_{{{{\mathrm{YSR}}}},{{{\mathrm{norm}}}}}(\vec r_{{{\mathrm{t}}}})$ (bottom) along a Mn_15_ chain recorded with an Fe-functionalized Nb tip. Parameters: *V*_stab_ = −10 mV, *I*_stab_ = 2 nA, *V*_mod_ = 100 µV, *B*_z_ = +0.5 T. The white scale bar corresponds to 1 nm and is the same for both panels. Source data

**Extended Data Fig. 3:**
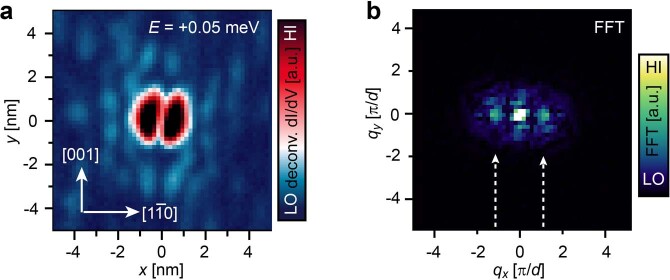
Experimental estimation of ${{{\boldsymbol{k}}}}_{{{{\mathbf{F}}}},0}$. **a**, Deconvoluted d*I*/d*V* map around a single Mn atom taken at $E = E_{\delta , + }$ = 0.05 meV. Parameters: *V*_stab_ = 6 mV, *I*_stab_ = 3 nA, *V*_mod_ = 40 µV. **b**, Absolute values of the Fourier transform (FFT) of the map in **a** with the central 2 × 2 nm^2^ of the image being suppressed using a Tukey window function to avoid FFT artifacts. The white arrows highlight the features around $2k_{\mathrm{F}}=q \approx \pm 1.2\pi /d$. Source data

We use scanning tunnelling microscopy (STM) and scanning tunnelling spectroscopy (STS) with a superconducting tip to probe the local density of states (LDOS) at subgap energies (Methods). Single Mn atoms on clean Nb(110) induce multiple YSR states (Fig. 1a)^23,33^. Their spatial anisotropy facilitates different hybridizations of the YSR states stemming from neighbouring Mn atoms by tailoring the directionality of nanostructures on the Nb(110) surface, as it has been shown for dimers of Mn atoms^33^. In particular, the lowest-energy YSR state (referred to as *δ* hereafter) extends along the [$1\bar 10$] direction (Fig. 1b–d). Thus, we construct chains along the [$1\bar 10$] direction (Fig. 2a). We expect this orientation to lead to a dominant hybridization of the lowest-energy *δ*-YSR states compared with the weaker coupling of all the other higher-energy YSR states, such as the state labelled as *α*_+/–_ in Fig. 1a. This is reasonable, especially because the interatomic distance in this configuration is large (*d* = 0.467 nm) compared with the interatomic distances for chains along [001] or $[1\bar 11]$. Similarly, Mn is in the centre of the transition metal series and its *d* states are energetically located at very high energies away from *E*_F_. Even when hybridizing, the bandwidth of the emerging *d* bands is expected to be too small to reach *E*_F_. Thus, ideally, the low-energy band structure would be reduced to an effective one-YSR-band system around *E*_F_, in contrast to the case elsewhere^23^ (Extended Data Fig. 1b and Supplementary Note 1). In this case, sufficient hybridization between the *δ* states results in a topologically non-trivial band structure irrespective of the model parameters, strongly reminiscent of the seminal Kitaev chain model for topological superconductivity^1^. The magnetic moments in Mn chains along the [$1\bar 10$] direction are ferromagnetically aligned (Extended Data Fig. 2 and Supplementary Note 2), thus providing all the necessary ingredients for topological superconductivity in the presence of any non-vanishing effective Rashba SOC *k*_h_. With the use of model calculations approximating the effective low-energy theory of a 1D chain of dilute YSR impurities^9^ (Methods and Supplementary Notes 3 and 4), we discuss the expected topological properties of chains crafted from single YSR atoms. Within this model, the chain is embedded in a three-dimensional superconductor. We additionally find very similar results using a tight-binding model for a chain on the surface (Supplementary Note 5). We start by modelling the *δ*-YSR states of a single Mn impurity^23,33^ (Methods and Fig. 1a) and use the same parameters to extrapolate to the case of a YSR chain. The resulting phase diagram shown in Fig. 1e demonstrates that the chain is indeed almost always in a topologically non-trivial phase^9^. This holds as long as the chain is sufficiently dilute to remain in an effective one-band scenario where only the hybridizing *δ*-YSR states are relevant and as long as the effective coherence length *ξ* of the substrate is not unrealistically small or its Fermi wavevector *k*_F,0_ has a very specific size. Experimentally, we can determine *k*_F,0_ to be (0.6 ± 0.1)π/*d* (Extended Data Fig. 3 and Supplementary Note 6), which is far from these critical points.

### End states and their energy splitting

To experimentally realize this concept, we construct Mn_*N*_ chains consisting of *N* atoms along the [$1\bar 10$] direction (Fig. 2a) by the controlled lateral manipulation of Mn atoms on the Nb surface using the STM tip (Methods). In Fig. 2b, we present an example of a Mn_32_ chain. In Fig. 2c, we show the spatially resolved deconvoluted differential conductance (d*I*/d*V*) maps around the chain. We find states at zero energy that are well localized at the chain’s ends with additional small LDOS oscillations in the interior of the chain. In contrast, energetically higher states (0.5 < |*E*| < 1.5 meV), which are most probably the bands derived from hybridizing *α*-YSR states, are distributed all over the chain (Extended Data Fig. 1a and Supplementary Note 1). Spectra from the dataset in Fig. 2c measured at the chain’s end and centre as well as on the bare Nb substrate (Fig. 2d) reveal a narrow zero-energy peak in the d*I*/d*V* signal localized on the chain’s end, corresponding to the zero-energy state of Fig. 2c. Peaks corresponding to the finite-energy states in Fig. 2c are distributed over the entire chain. Such a clearly resolved zero-energy end state is typically considered as an indication for MMs^11–18^.

**Fig. 3:**
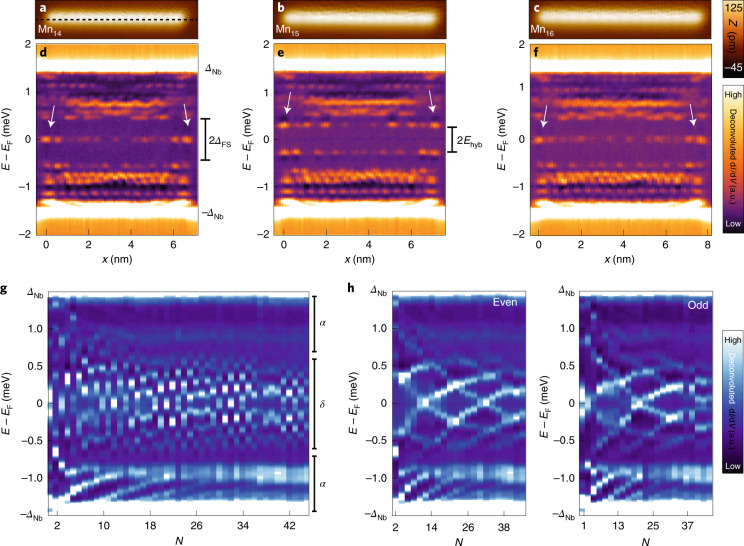
Chain-length dependence of in-gap states. **a**–**c**, STM images of Mn_*N*_ chains with *N* = 14 (**a**), 15 (**b**) and 16 (**c**). **d**–**f**, Corresponding deconvoluted d*I*/d*V* line profiles acquired along the longitudinal axis through the centre of the three chains (as indicated by the dashed line along the Mn_14_ chain). The STM images are aligned with the d*I*/d*V* line profiles and edge states are highlighted by white arrows. **g**, Sequence of d*I*/d*V* spectra obtained on the end of Mn_*N*_ chains for different *N* values located at a different sample position as the chains in **a**–**f**. The spectral features associated with the bands from hybridizing *α*- and *δ*-YSR states are marked on the right side (Extended Data Fig. 1). **h**, Dataset from **g** with chains of even and odd *N* plotted in separate panels. Source data

Since we construct the chains in an atom-by-atom manner, we are able to track changes in the low-energy states for each length *N* and to probe the robustness of the zero-energy end state. As an example, we show the deconvoluted d*I*/d*V* signal along the chain in a 1D line of spectra (called the d*I*/d*V* line profile hereafter) for *N* = 14–16 (Fig. 3a–f). Interestingly, we find similar zero-energy end states as in Fig. 2c for *N* = 14 and 16 (Fig. 3d,f, arrows), separated from the higher-energy states by a large gap *Δ*_FS_ = 400 µeV. Instead, for *N* = 15, there are two states with a similarly strong localization on the chain’s ends (Fig. 3e, arrows) but split by *E*_hyb_ ≈ 300 µeV symmetrically around *E*_F_. Importantly, this shows that the two end modes on both sides are a single, coherent quantum state of the chain, since their energies on both ends of the chain are intertwined. As substantiated later, these states can be interpreted as PMMs with a residual MM coupling due to the finite length of the chain. If this is the case, their coupling will not only depend on the length of the chain but also on the wavefunction modulation of the PMMs.

To investigate this effect, we show the deconvoluted d*I*/d*V* signal measured at the end of another, structurally identical chain with varying chain length *N* (Fig. 3g). With increasing *N*, we added Mn atoms to one chain end and measured the d*I*/d*V* spectra at the opposite end to trace the states’ energies. In accordance with Fig. 3d–f, we find that the energy of the state closest to *E*_F_, which corresponds to the end state, is modulated with a period of Δ*N* ≈ 2. This trend continues up to the longest chains built by us (*N* = 45). The remaining density of oxygen impurities on the surface limits the maximum length of ordered magnetic chains to this length, corresponding to 20–25 nm. The modulation effect is the most apparent when we separately plot the chains with even and odd *N* (Fig. 3h). Here the change in the subgap state energies appears to be continuously changing as a function of *N*. Most notably, for certain chain lengths, such as *N* = 12, 21, 32 and 42, the energy of the end state can be tuned to zero within the experimental peak width, which corresponds to Δ*E* = 50 µeV. In contrast to this observation, the hybridizing *α*-YSR states evolve into a comparably narrow band (Fig. 3g), which is irrelevant for the topological properties of the system (Extended Data Fig. 1b and Supplementary Note 1).

### Theoretical modelling of length-tunable chains

**Fig. 4:**
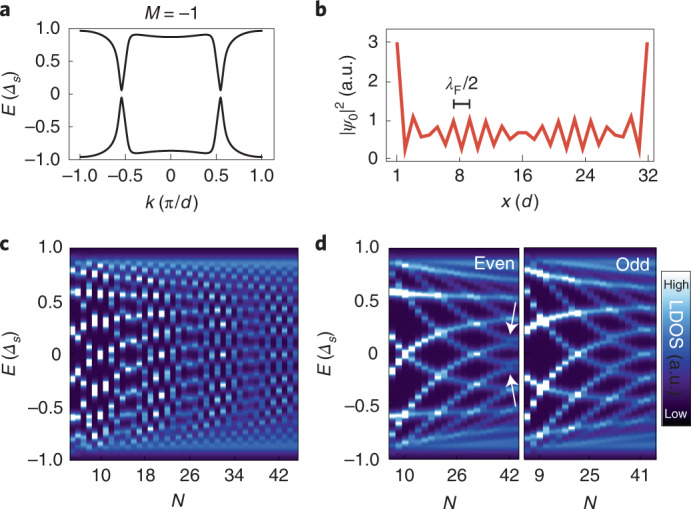
Theoretical model for PMMs derived from YSR bands. **a**, Band structure of an infinite chain of YSR impurities using parameters describing the experimentally measured energy and particle–hole asymmetry of the *δ*-YSR states. The topological index of the infinite chain is non-trivial (*M* = −1). **b**, Calculated LDOS of the state with least energy along a finite chain of 32 sites using the parameters from **a**. **c**, LDOS on the first site of a finite chain of length *N*. **d**, Dataset from **c** with chains of even and odd *N* plotted in separate panels. The arrows exemplarily show a position where weak anticrossings of the states are visible, indicating the presence of *p*-wave superconducting pairing (Supplementary Fig. 2). Parameters: *A* = 1.1, *B* = 0.2, *k*_h_ = 0.05π/*d*, *k*_F,0_ = 0.53π/*d*, *ξ* = 4.67 nm, *d* = 0.467 nm and *Δ*_*s*_ = *Δ*_Nb_ = 1.5 meV (Methods and Supplementary Note 3). Source data

To substantiate that the observed end states are indeed PMMs from the two ends of the chain, we performed the aforementioned model calculations^9^ to simulate the chains of *N* sites in contact with a superconducting host. We emphasize that within this model, we are unable to explain the experimental data in Fig. 3g,h when assuming a topologically trivial phase. Yet, we find regimes of the model in the topologically non-trivial phase qualitatively reproducing the experimental data on finite chains (Fig. 4). Using the parameters yielding the band structure of the YSR chain in Fig. 4a, we find end states at zero energy with a strong localization at the terminal sites for special lengths of the chain (Fig. 4b). Most notably, the Δ*N* ≈ 2 modulation of the low-energy states is in good agreement with the experiment (Figure 3g,h and Fig. 4c,d). The modulation turns out to be induced by a particular position of the Fermi points in the low-energy band structure: since the YSR band crosses *E*_F_ at *k*_F_ ≈ ±π/2*d*, the Fermi wavelength *λ*_F_ = 2π/*k*_F_ ≈ 4*d* is specifically related to the lattice constant. This leads to a modulation of eigenenergies in chains of length *N* with Δ*N* ≈ 2. This type of beating effect in a quantum-size-limited system has been observed on other platforms, for example, quantum well states in thin films of Pb/Si(111) (refs. ^34–36^) or in predictions for Andreev-bound states in superconducting carbon nanotubes^37^. Equally, the effect can be understood in terms of PMMs: it has been shown that MMs feature an LDOS modulation with *λ*_F_/2 (ref. ^38^) (Fig. 4b). Accordingly, the overlap, and thus the interaction of MM wavefunctions from both ends of the chain, is expected to oscillate with the total chain length with Δ*N* ≈ 2. Notably, the approximate zero energy of the MM for particular chain lengths is not protected by the topological properties of the electronic band structure but is tuned by the atomically precise experimental control of the chain length. Within the model, they evolve into isolated MMs for very long chains (Supplementary Notes 3 and 4) and can thus be seen as their precursors^19–21^. The large gap *Δ*_FS_ to higher-energy excitations can be interpreted as a finite-size gap resulting from the steep YSR band (Fig. 4a).

The agreement with our model calculations indicates that the relevant single band is indeed formed by hybridizing *δ*-YSR states of the single Mn atoms expanded along the [$1\bar 10$] direction, which have energies close to zero for isolated atoms (Fig. 1a)^23,33^.

### Exclusion of other topologically trivial causes

The question is whether other topologically trivial explanations for the observed end states exist. The emergence of trivial zero-energy states due to disorder effects is frequently discussed for various Majorana platforms^22,39–41^. We can rule out this explanation because of the geometrically perfect structure of our chains. Most importantly, the fact that the end modes at both ends of the chain change equally when perturbing only one side (Fig. 3d–f and Supplementary Note 8) proves that the end state is a collective mode of the 1D structure. We can, therefore, rule out that the end states are zero-dimensional features induced by local defects or localized YSR states^24–27^. The observation of this correlation between both ends is a key advantage compared with previous experimental studies of potential topological superconductors, where only one end of a nanowire is probed^11–16,20^. It is possible that the localization of the wavefunction closest to *E*_F_ is less pronounced than the experiment suggests (Supplementary Notes 3 and 4). Especially since both YSR states and MMs can be predominantly located in the superconducting host, the measurement of LDOS above the atomic chain could suppress the signal in the chain’s interior and amplify the intensity at the chain’s ends^14^. Note that the topological phase of the infinite system would still be non-trivial in this case. Topologically trivial phases could only be compatible with the experiment in the presence of additional low-energy bands. In this scenario, an even number of MMs from different bands would inevitably interact for arbitrarily long chains, thereby lifting their degeneracy and destroying topological protection. Experimentally, all the features from additional bands are well separated from *E*_F_ (Fig. 3g, Extended Data Fig. 1b and Supplementary Note 1), providing strong evidence that our chains indeed realize an effective one-band model in the low-energy limit. As such, our model calculations reveal that the system is topologically non-trivial in the relevant parameter regime (Fig. 1e and Supplementary Note 3).

It is important to note, however, that the MMs in an infinite system only experience a topological protection of the size of the bulk topological gap *Δ*_*p*_. The topological gap for the system at hand is calculated to be 50 µeV (Supplementary Note 7), which is considerably smaller than the observed energy splitting of the PMMs *E*_hyb_ and the finite-size gap *Δ*_FS_ in our experimentally realized chains (Fig. 3, Supplementary Note 3 and Supplementary Fig. 1e). For systems with this sequence of orders of magnitude of the different parameters, the *p*-wave pairing *Δ*_*p*_ manifests as an emergent apparent avoided crossing of the lowest- and second-lowest-energy states at positions exemplarily indicated by the arrows in Fig. 4d, which is just too small to be detected within our experimental energy resolution (Supplementary Figs. 1 and 2 show the evolution of the avoided crossings in longer chains). The long-range extension of MMs has been shown to be inversely related to *Δ*_*p*_ (ref. ^42^). Our results indicate that the observation of a well-localized zero-energy end state in a finite-size topological superconductor does not directly imply that the corresponding MMs are non-interacting under the influence of small perturbations (Supplementary Note 4). We expect the energy of the end modes to converge to energies below *Δ*_*p*_ only for long chains with *N* > 70 corresponding to chain lengths of 35 nm (Supplementary Figs. 1 and 2). However, the interactions of the observed fine-tuned zero-energy PMMs with the continuum of 1D modes are strongly suppressed by the presence of a relatively large finite-size gap *Δ*_FS_.

## Conclusions

One way to improve the localization further and to reduce the interactions of MMs would be to enhance the Rashba SOC in the system, either using a different superconducting host, heavy-material interlayers or artificial SOC^5,43–45^, all of which are expected to enhance *Δ*_*p*_. However, as shown in the topological phase diagram in Fig. 1e using the parameters from Fig. 4 (black dashed lines), although the system is deep in the topological phase, it is near a gap closing at *k*_F_ ≈ ±π/2*d* (Supplementary Note 3). Note that, uncommonly, this gap closing is between two topologically non-trivial regions^9^. Despite constant SOC, this gap closing adds a previously disregarded constraint for realizing strongly protected MMs in a hard gap *Δ*_*p*_ in future experiments. Moreover, even if the hard gap of the infinite system cannot be experimentally resolved, a gradual separation of the lowest-energy states from the continuum (Supplementary Fig. 2) could serve as an indicator for the magnitude of *Δ*_*p*_ and the degree of topological protection.

**Fig. 5:**
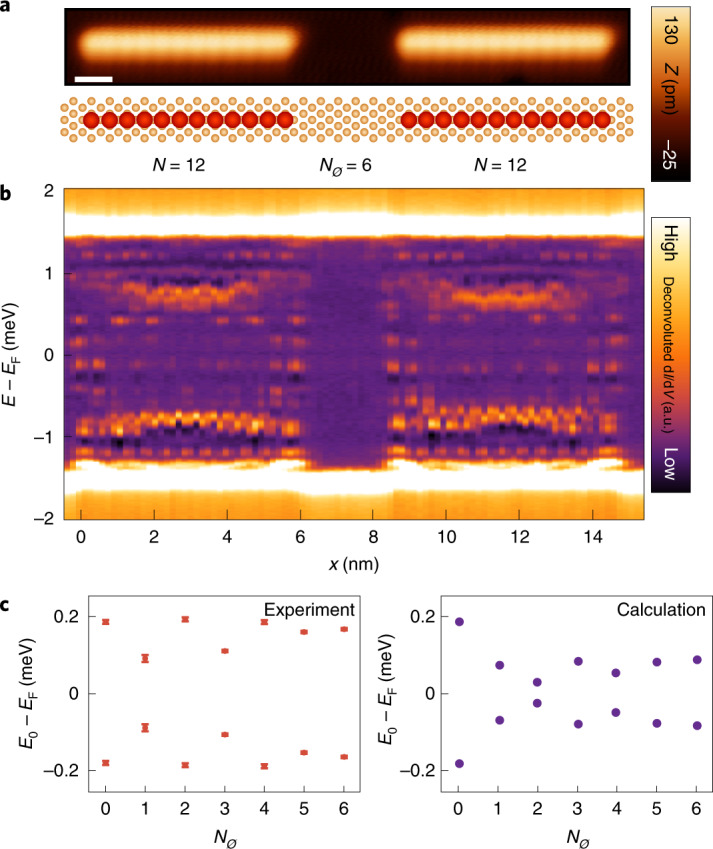
Junctions of interacting Mn_12_ chains. **a**, STM image (top) of two Mn_12_ chains with *N*_∅_ = 6 empty sites between the chains and a respective sketch of the geometric structure (bottom). Scale bar, 1 nm. **b**, d*I*/d*V* line profiles along the longitudinal axes through the centres of both chains in **a** aligned with the topography. **c**, Experimentally measured (left) and calculated (right) energy *E*_0_ of the lowest-energy state versus interchain spacing *N*_∅_. The same model parameters as in Fig. 4 are used. The error bars on the values of *E*_0_ are standard errors resulting from a least-square fit of two Gaussian peaks to the d*I*/d*V* spectra on the left end of the left chain. Source data

**Extended Data Fig. 4:**
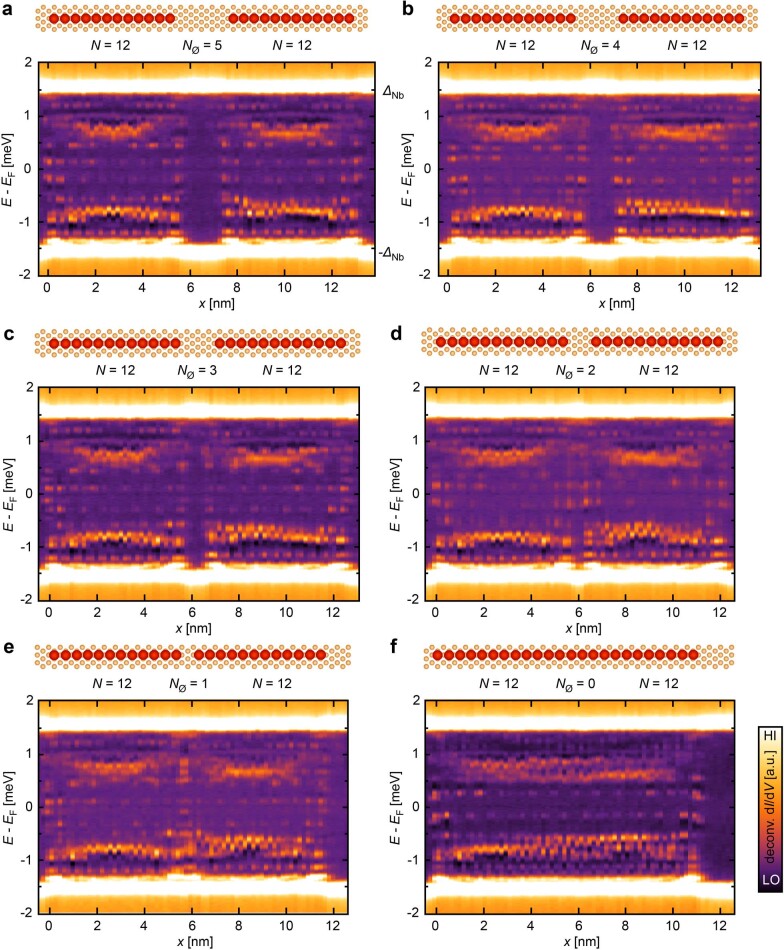
Interacting Mn_12_ chains. **a**, Geometric structure and experimental d*I*/d*V* line-profiles along two Mn_12_ chains with $N_\emptyset$ = 5 empty sites between the chains. **b**, The same measurement for $N_\emptyset$ = 4. **c**, $N_\emptyset$ = 3. **d**, $N_\emptyset$ = 2. **e**, $N_\emptyset$ = 1. **f**, $N_\emptyset$ = 0, that is a merged Mn_24_ chain. Parameters: *V*_stab_ = −6 mV, *I*_stab_ = 1 nA, *V*_mod_ = 20 µV. Source data

Owing to the atomic-scale control of nanostructure fabrication by single-atom manipulation, we can envision studies of MM interaction in artificially created networks of interacting chains^46–48^. An example for a junction of two Mn_12_ chains with varying interchain distance is shown in Fig. 5a,b. We can control the number of unoccupied adsorption sites *N*_∅_ with the precision of a single atom and analyse the energy of the state closest to *E*_F_ (Fig. 5c, left, Extended Data Fig. 4 and Supplementary Note 8 show the full dataset). Its energy modulates with Δ*N*_∅_ ≈ 2 again, providing evidence that the two chains are coupled through long-range interactions of the YSR states with a strength oscillating with *λ*_F_/2. This oscillatory change in the energy of the end state with interchain distance is qualitatively consistent with model calculations using the model and parameters from Fig. 4 and removing *N*_∅_ lattice sites of the YSR chain (Fig. 5c, right). However, a quantitative description of these interactions will require more advanced modelling. We emphasize that the observed energy splitting is an interplay of inter- and intrachain PMM interactions, where the latter is discussed above for single chains. Building similar networks of sufficiently long chains, where the MMs on each individual chain are initially at *E*_F_ and topologically protected by gap *Δ*_*p*_, may ultimately enable to explore the theoretically predicted robustness of such MMs under local perturbations acting only on one end of the chain.

## Methods

### Experimental procedures

All the experiments were performed in a home-built STM facility operated at a temperature of *T* = 320 mK (ref. ^49^). We used a Nb(110) single crystal as a substrate, cleaned by high-temperature flashes to *T* > 2,700 K. Using this cleaning procedure, atomically clean surfaces with only few residual oxygen impurities on the surface can be obtained^50^. Subsequently, single Mn atoms were deposited onto the cold surface (*T* < 7 K), resulting in a statistical distribution of adatoms. Superconducting tips were created by indenting electrochemically etched W tips into the substrate, thereby picking up a large cluster of superconducting Nb. STM images were measured maintaining a constant tunnelling current *I* when applying a constant bias voltage *V*_d.c._ across the tunnelling junction. For the measurement of the differential tunnelling conductance (d*I*/d*V*) spectra, the tip was stabilized at bias voltage *V*_stab_ and current *I*_stab_. Subsequently, the feedback loop was opened and the bias voltage was swept from –4 to +4 mV. The d*I*/d*V* signal was measured using a standard lock-in technique with a small modulation voltage *V*_mod_ (r.m.s.) of modulation frequency *f* = 4.142 kHz added to *V*_d.c._. The d*I*/d*V* line profiles and maps were acquired, recording multiple d*I*/d*V* spectra along a line or grid, respectively. All the datasets shown in the main manuscript were measured using *V*_stab_ = *V*_d.c._ = −6 mV, *I*_stab_ = 1 nA and *V*_mod_ = 20 µV. Note that at these stabilization parameters, the contribution of Andreev reflections can be neglected (Supplementary Note 9). Superconducting Nb tips have been chosen to increase the effective energy resolution. The measured differential tunnelling conductance d*I*/d*V* is, thus, proportional to the convolution of the LDOS of the sample and the density of states of the superconducting tip. We show the numerically deconvoluted STS data throughout the manuscript, resembling the sample’s LDOS (Supplementary Note 9). The chains were assembled using lateral atom manipulation^51^ at low tunnelling resistances of *R* ≈ 30–60 kΩ.

### Model for single and hybridizing YSR states

The theoretical analysis of the single and hybridized YSR states follows ref. ^23^, adapting it to the material-specific parameters. We repeat the essential definitions here to be self-consistent. In the model of single YSR impurities embedded in a superconducting host (as described elsewhere^9,52^), the subgap states are characterized by a magnetic scattering term *J* and an additional non-magnetic scattering term *V*. Their energy *E*(*A*, *B*) and particle weight *P*(*A*, *B*) can be written in terms of the dimensionless parameters *A* = π*ν*_0_*J* and *B* = π*ν*_0_*V*, with the normal-phase density of states *ν*_0_ and the superconducting *s*-wave pairing *Δ*_*s*_.

1 $$\begin{array}{*{20}{c}} {E\left( {A,B} \right) = \varDelta _{{{{s}}}}\frac{{1 - A^2 + B^2}}{{\sqrt {\left( {1 - A^2 + B^2} \right)^2 + 4A^2} }}} \end{array}$$

2 $$\begin{array}{*{20}{c}} {P\left( {A,B} \right) = \frac{{1 + \left( {A + B} \right)^2}}{{1 + \left( {A + B} \right)^2 + 1 + \left( {A - B} \right)^2}}} \end{array}$$

The particle weight *P*(*A*, *B*) determines the ratio between the observed peak heights of the positive- and negative-bias YSR peaks measured in the low-conductance regime of an STS experiment^23,53^. We find that the *δ*-YSR states of Mn atoms on Nb(110) (ref. ^33^) are well reproduced by choosing *A* = 1.1 and *B* = 0.2, leading to the correct energy and particle–hole asymmetry of the experimentally measured peaks in d*I*/d*V* (Fig. 1a shows the fit with two Gaussians). Accordingly, these parameters are used for the description of chains. Note that the choice of *A* = 0.94 and *B* = –0.20 also reproduces the YSR peaks well and leads to very similar topological phase diagrams.

To describe chains of weakly interacting YSR atoms, we use a model based on another work^9^, which is extended to include non-magnetic scattering at the YSR impurity (that is, the *B* term in equations (1) and (2)). For details on this model and its derivation, we refer to another study^23^. The low-energy Bogoliubov–de Gennes Hamiltonian for the YSR chain is characterized by the scattering parameters *A* and *B* of the single YSR states, the Fermi wavevector of the superconducting host in the metallic state *k*_F,0_, the effective coherence length in the YSR chain *ξ* and an effective Rashba SOC parameter *k*_h_. One can approximately relate *k*_h_ to the usual definition of the Rashba SOC parameter via $\hbar {\lambda} \approx \frac{{\Delta}_s {\xi} k_{\mathrm{h}}}{k_{{\mathrm{F}},0}}$, where ℏ is the reduced Planck constant. We compute the LDOS as a function of energy *E* and position *x* along a 1D lattice of *N* sites (Fig. 4) by diagonalizing the low-energy Hamiltonian (given elsewhere^23^) and summing over all the pairs of eigenvalues *E*_*i*_ and eigenvectors *ψ*_*i*_:

3 $$\begin{array}{lll} {{\mathrm{LDOS}}\left( {E,x} \right)} &=& {\mathop {\sum }\limits_i \left[ {P(A,B)\left| {\psi _{i,\mathrm{e}}(x)} \right|^2 + (1 - P({{{\mathrm{A}}}},B))\left| {\psi _{i,\mathrm{h}}(x)} \right|^2} \right]} \\ & & \times{\left( { - \frac{{\partial f(E - E_i,\,T = 320\;{{{\mathrm{mK}}}})}}{{\partial E}}} \right)}, \end{array}$$

with the respective particle (e) and hole (h) components of the solutions and the Fermi–Dirac distribution function *f*(*E*, *T*) simulating the experimental thermal broadening. In order to accurately obtain the particle–hole asymmetry of all the states in terms of the physically original quasiparticles in equation (3) (which is measured in the experiment), *P*(*A* = 1.1, *B* = 0.2) is multiplied with the particle component of a state and (1 – *P*(*A* = 1.1, *B* = 0.2)) is multiplied with the hole component. We obtain the band structure for an infinite chain by Fourier transformation of the Hamiltonian with periodic boundary conditions applied^9^. For the numerical calculations shown in Fig. 4, we used the parameters *A* = 1.1, *B* = 0.2, *k*_h_ = 0.05π/*d*, *k*_F,0_ = 0.53π/*d*, *ξ* = 4.670 nm, *d* = 0.467 nm and *Δ*_*s*_ = 1.5 meV. Using these values, we estimate a Rashba SOC parameter of $\hbar {\lambda} \approx \frac{{\Delta}_s {\xi} k_{\mathrm{h}}}{k_{{\mathrm{F}},0}}$ = 0.0066 eV Å, which is in a reasonable range for the experimental system in this work compared with values in the literature^54^, considering the relatively weak SOC in Nb. The value for *k*_F,0_ is—within the error bar—compatible with the experimentally determined value (Supplementary Note 6). Note that the only free parameters that cannot be directly determined experimentally from single YSR states are *k*_h_ and *ξ* (Supplementary Note 3).

The topological invariant *M* is calculated as

4 $$\begin{array}{*{20}{c}} {M = \mathrm{sgn}\left\{ {{{{\mathrm{Pf}}}}\left[ {\tilde H\left( 0 \right)} \right]{{{\mathrm{Pf}}}}\left[ {\tilde H\left( \uppi \right)} \right]} \right\}} \end{array},$$

where Pf denotes the Pfaffian and $\tilde H\left( k \right)$ is the *k*-space Hamiltonian in the Majorana basis^1^.

## Online content

Any methods, additional references, Nature Research reporting summaries, source data, extended data, supplementary information, acknowledgements, peer review information; details of author contributions and competing interests; and statements of data and code availability are available at 10.1038/s41565-022-01078-4.

## Supplementary information

Supplementary Information Supplementary Figs. 1–8, Notes 1–9, additional theoretical simulations and Discussion.

## Data Availability

Source data are provided with this paper. All other data supporting the findings of this study are available from the corresponding author upon reasonable request.

## Source data

Source Data Fig. 1 Statistical source data of Fig. 1a–e in separate .txt files.
Source Data Fig. 2 Statistical source data of Fig. 2b–d in separate .txt files.
Source Data Fig. 3 Statistical source data of Fig. 3a–h in separate .txt files.
Source Data Fig. 4 Statistical source data of Fig. 4a–d in separate .txt files.
Source Data Fig. 5 Statistical source data of Fig. 5a–c in separate .txt files.
Source Data Extended Data Fig. 1 Statistical source data of Extended Data Fig. 1a,b in separate .txt files.
Source Data Extended Data Fig. 2 Statistical source data of Extended Data Fig. 2a,b in separate .txt files.
Source Data Extended Data Fig. 3 Statistical source data of Extended Data Fig. 3a,b in separate .txt files.
Source Data Extended Data Fig. 4 Statistical source data of Extended Data Fig. 4a–f in separate .txt files.

## Code availability

Software codes supporting the findings of this study are available from the corresponding author upon reasonable request.
