# Supplementary material for: Precursors of Majorana modes and their length-dependent energy oscillations probed at both ends of atomic Shiba chains
Source: Nat Nanotechnol. 2022 Mar 7;17(4):384–9. doi: 10.1038/s41565-022-01078-4 (PMC9018407; doi:10.1038/s41565-022-01078-4)
Supplement: Supplementary file 1 — Supplementary Figs. 1–8, Notes 1–9, additional theoretical simulations and Discussion. [file 41565_2022_1078_MOESM1_ESM.pdf]

nature portfolio

https://doi.org/10.1038/s41565-022-01078-4

Supplementary information

Precursors of Majorana modes and their length-dependent energy oscillations probed at both ends of atomic Shiba chains

In the format provided by the authors and unedited

Precursors of Majorana modes and their length-dependent energy oscillations probed at both ends of atomic Shiba chains

Supplementary Information

Lucas Schneider, Philip Beck, Jannis Neuhaus-Steinmetz, Levente Rózsa, Thore Posske, Jens Wiebe and Roland Wiesendanger

Correspondence to: jwiebe@physnet.uni-hamburg.de

Supplementary Note 1 | Justification of an effective single YSR band system

The analysis presented in the main text focuses on a single YSR band. The good agreement of the low-energy data in Fig. 3 with the one-band model in Fig. 4 already suggests that we have indeed effectively realized a one-band system. However, since the presence of additional bands can influence the topological properties of the hybrid system (see, e.g., Ref. 1), we discuss the possible existence of other YSR bands.

Recently, the analysis of quasiparticle interference (QPI) in YSR chains was shown to reveal information about the sub-gap band structure of the chains[1]. The sub-gap Bogoliubov-de-Gennes quasiparticles in a magnetic chain experience strong confinement in the chain. Therefore, states with momentum k_{i} are scattered to momenta $k_{\mathrm{f}} = -k_{\mathrm{i}}$ with high probability. The interference between k_{i} and k_{f} leads to a modulation in the local density of states with $q = |k_{\mathrm{i}} - k_{\mathrm{f}}|$. From the respective scattering vector q and energy E, we are able to relate energy and momentum for a number of states, thus gaining information about the band dispersion[1–3].

We can apply this method to the spectroscopic data presented in the main text. In Extended Data Fig. 1a, we provide additional dI/dV maps similar to the ones of Fig.2c of the main text but acquired at larger energies much closer to the gap edge Δ_{Nb} of the substrate. They reveal additional states with an integer number of maxima n_{α}. These states are related to other multiorbital YSR states, most probably the α-YSR state[1,4], which is different from the δ-YSR state investigated in the main text, as we will substantiate in the following. In order to extract the dispersions of all sub-gap bands, we calculate the line-wise absolute squares of the Fourier transformations of dI/dV line-profiles similar to Fig. 3d-f of the main text. We sum over Mn_N chains of various lengths from $10 \leq N \leq 32$ to collect as many points as possible. The result can be seen in Extended Data Fig. 1b. Most notably, close to E_{F}, the FFT is dominated by a single faint band crossing E_{F} at $k_{\mathrm{F}} \approx \pm\, \pi/2d$, which we can assign to the δ-YSR band. This again supports the single-band low-energy model described in the main text and the choice of the Fermi wavevector for this band. All other dispersing structures remain at higher energies. In particular, the states shown in Extended Data Fig. 1a belong to a relatively flat band that stays close to the gap edge Δ_{Nb} as it can be seen at energies 0.5 meV $< |E| <$ 1.5 meV in Extended Data Fig. 1b. Most probably it derives from hybridization of the α-YSR states as can be seen in Fig. 3g of the main text, where the features within this energy range gradually emerge from the α-YSR state of the individual atoms (cf. Fig. 1a of the main text) with increasing chain length. Moreover, these features have a similar particle-hole asymmetric spectral weight as the single atom α-YSR state. These states are also responsible for the clear band formation in Mn chains on Nb(110) along the [001] direction[1] where this band crosses the Fermi level and opens a topological gap in contrast to the chains along the $[1\bar{1}0]$ direction investigated here. It is plausible that the bandwidth of this band is reduced in chains along $[1\bar{1}0]$ with respect to the [001] chains, since the inter-atomic distance is enhanced with respect to that in the chains along [001]. Since this band is confined to an energy window of 0.5 meV $< |E| <$ 1.5 meV (cf. Extended Data Fig. 1b and Fig. 3g of the main text) and thus well separated from E_{F} it does not contribute to the topological classification of the chains along $[1\bar{1}0]$ within a low-energy effective model. Thus, by choosing a different chain geometry, we were able to controllably remove the influence of the α-YSR band on the low-energy physics of our atomic chains.

In addition to the δ- and α-YSR states discussed in the main manuscript text and here, there are two other additional less intense YSR states of the single Mn atom[1,4], see e.g. the weak resonances at ±0.8 meV in Fig.1a. Similar to the α-YSR states, these additional YSR states can be disregarded for the low-energy effective model here for two reasons. Firstly, they are much farther away from E_{F} as compared to the δ-YSR state. Secondly, unlike the δ-YSR state, they do not have lobes along $[1\bar{1}0]$. Therefore, in chains built along $[1\bar{1}0]$, these additional states aren't expected to hybridize sufficiently strong to form Shiba bands which cross E_{F}.

Supplementary Note 2 | Magnetic structure of the chains

In Extended Data Fig. 2a, we show dI/dV maps of a Mn_{11} chain along $[1\bar{1}0]$ obtained with a spin-polarized Cr tip. While the tip magnetization has been shown to be stable using a magnetic reference chain presented in Ref. 5, the dI/dV signal on the chain changes with an applied out-of-plane field B_{z}. This indicates that the chain's magnetization aligns with B_{z}. We find a homogeneous contrast along the chain, indicating a ferromagnetic alignment of the magnetic moments. Furthermore, we repeated this measurement using a superconducting Nb tip functionalized with Fe atoms, inducing YSR states on the tip. We can exploit the spin-polarization of the YSR states to map the magnetic structure of our sample[5]. The result is shown in Extended Data Fig. 2b, measured on

a Mn_{15} chain along $[1\bar{1}0]$. The YSR-asymmetry parameter $A_{\mathrm{YSR,norm}}(\vec{r}_{\mathrm{t}})$ at the tip position $\vec{r}_{\mathrm{t}}$ is defined in Ref. 5 and resembles the sample's spin-polarization. Again, we find a finite and spatially homogeneous spin-contrast, confirming the ferromagnetic structure of the chains. The observation that the magnetization can be aligned by a relatively small out-of-plane magnetic field further indicates that the easy axis of the chain is perpendicular to the surface.

Supplementary Note 3 | Additional model calculations for YSR chains

Studying the model introduced in the Methods section of the main text, we find that with the parameters used in Fig. 4 of the main text, the chain is deep in a topological phase, although with a comparably small topological gap of $\Delta_p = 80$ µeV (see Fig. 1e of the main text). Thus, we expect isolated MMs to form at the chains' ends for $N \to \infty$. We study this limit theoretically in Supplementary Fig. 1.

In Supplementary Fig. 1a, we plot the energy resolved LDOS along a chain of N = 200 using the parameters from Fig. 4 of the main text. This shows that the exponential decay length of the Majorana wavefunction is long, which explains their significant residual interaction in experimentally accessible chains of N < 50. In Supplementary Figs. 1b,c, we show the extended plot from Figs. 4c,d of the main text up to chain lengths of N = 89. For N > 70, a clear separation of the MMs from the bulk states can be observed. Furthermore, we can see how the PMMs at chain lengths considered in the experiment are connected to the isolated MMs in the infinite chain by following the low-energy lines in Supplementary Fig. 1c. The temperature broadening was reduced to an equivalent of $T = 200\ \mathrm{mK}$ in this plot for better visibility. In Supplementary Fig. 1d, we depict the lowest energy wavefunction for N = 200, clearly illustrating the exponential decay of the MM for long chains. Although the MM has significant weight especially on the chains' end sites, there is strong spectral weight spread over the first $\sim$70 sites. Furthermore, we can compute the MM energy splitting vs. chain length, as plotted in Supplementary Fig. 1e. As visible in the plot, the MMs converge to energies below the topological gap Δ_p, which is defined by the energy of the energetically lowest bulk state in infinite chains, for N > 70.

Note, however, that the decay constant strongly depends on the strength of the Rashba spin-orbit coupling (SOC) term k_{h}, which cannot be directly measured in the experiment. The effect of the Rashba SOC strength k_{h} is presented in Supplementary Fig. 2 for chains up to N = 180 sites. For the exact value k_{h} = 0.00, only conventional finite-size quantized bulk states are found to be filling the gap. Here, the topological index is not well defined since the system is entirely gapless. However, for any arbitrarily small k_{h}, the chain is in the topologically non-trivial phase. As it is visible in Supplementary Fig. 2, the lowest energy states separate more and more from the higher energy states with increasing k_{h}. This separation is even slightly visible for very small values (e.g. k_{h} = 0.01 π/d in Supplementary Fig. 2a), in agreement with the bulk-boundary correspondence. For larger k_{h}, the topological gap Δ_p increases and the convergence of the PMMs to zero energy is faster. Since we cannot directly determine the exact value of k_{h} from experimental data, conclusions about the chain length needed to observe isolated MMs are challenging to draw. However, we can rule out the case of k_{h} = 0.00, since previous studies of the Mn/Nb(110) system found that Rashba SOC in this system is significant and can induce observable effects like splittings of the YSR states in antiferromagnetic dimers[4] or gaps in a YSR band of a Mn chain[1]. Therefore, isolated MMs are clearly expected to occur in long Mn chains. The observations of Supplementary Fig. 2 are in agreement with the expected inverse relationship between Δ_p and the decay length of the Majorana wavefunction[6]: namely, for larger values of Δ_p, the modes converge faster towards zero-energy. This is further validated by analyzing the long-range decay of the wavefunction's envelope. For this, we fit the envelope of the lowest-energy wavefunction in a chain with N = 800 sites to the expression

$$\mathrm{LDOS}(0, x) = A \cdot (e^{-\kappa(x-1)} + e^{\kappa(x-800)}), \qquad (S1)$$

with the decay parameter κ and a normalization factor A. The result for the decay length κ^{-1} is shown in Supplementary Fig. 3b and clearly anti-correlates with the magnitude of the topological gap, i.e. κ^{-1} diverges when the gap closes and is small for large gaps. This further demonstrates that the topological nature of the end states manifests itself in the long-range decay of the wavefunction instead of the observed apparent localization on the terminal sites only.

Another interesting observation is the presence of avoided crossings between the lowest energy state and the higher energy excitations (Supplementary Fig. 2). The degree of separation from the trivial states increases with

k_h and could thus potentially serve as an estimator for the pairing magnitude Δ_p if the topological gap itself is not resolved in short chains with PMMs.

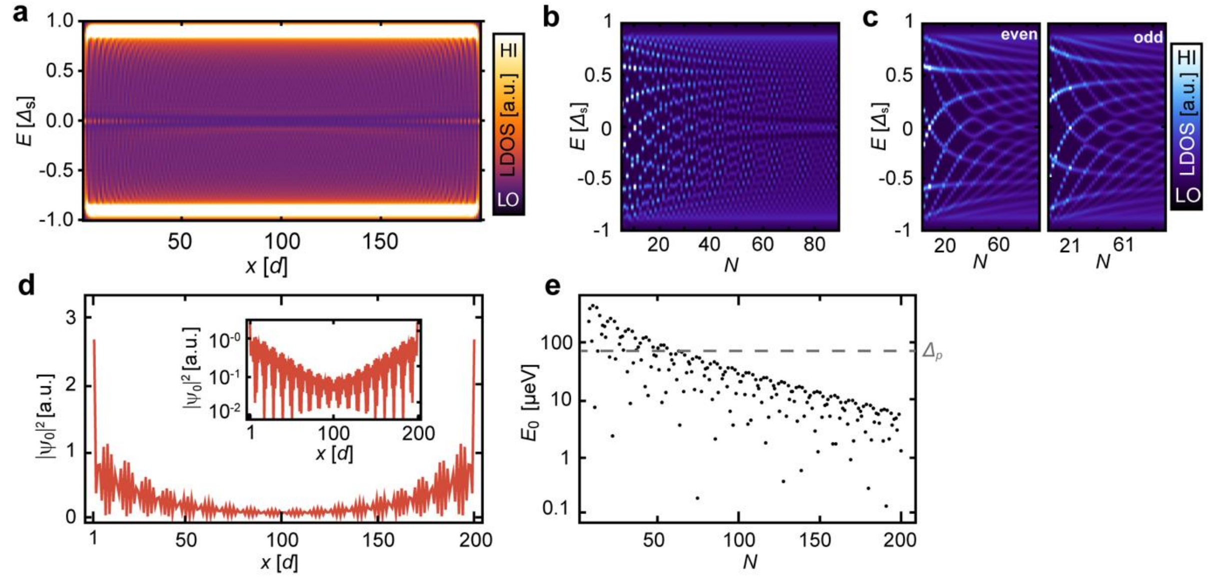

Supplementary Fig. 1 | Calculations for longer chains. a, Calculated LDOS along a finite chain of 200 sites. **b**, LDOS on the first site of a finite chain of length *N*. **c**, Dataset from **b** with chains of even- and odd-*N* plotted in separate panels. **d**, Majorana wavefunction for a chain of 200 sites. Inset: logarithmically scaled plot, showing the long-range exponential behavior of the decay. **e**, Energy of the lowest-energy state vs. chain length. The magnitude of the topological gap Δ_p for an infinite chain is indicated by the dashed line. For all simulations, the parameters from the main text were used.

In order to reduce the MM interactions in future experiments, it would thus be beneficial to enhance the topological gap Δ_p. Surprisingly, an increase of the Rashba SOC parameter k_h does not necessarily lead to an increase in Δ_p. This is shown in Supplementary Fig. 3a, where the system's gap is plotted against k_h and $k_{\mathrm{F},0}$. Supplementary Fig. 3b shows a line-cut through the gap diagram in Supplementary Fig. 3a at $k_{\mathrm{F},0} = 0.53\,\pi/d$, which was found to match the experiment. As already seen in Fig. 1e of the main text, there is a gap closing between two topologically non-trivial regions at $k_{\mathrm{F},0} \approx 0.5\pi/d$. The reason lies in the *k*-dependence of the *p*-wave pairing strength $\Delta(k)$ in our model, as it will be elaborated in the following. Interestingly, one finds that $\Delta(k)$ features zero crossings at $k = k_{\mathrm{F},0}$ in the case of vanishing non-magnetic scattering $B = 0$ [7]. Thus, no topological gap can be opened by the pairing term if the Fermi wavevector k_F defined by the crossing of YSR bands through E_F equals the Fermi wavevector of the host, i.e. $k_\mathrm{F} = k_{\mathrm{F},0}$. In Supplementary Fig. 3c, we show the *k*-dependent superconducting pairing $\Delta(k)$, and the *k*-dependent hopping terms h_k are shown in Supplementary Fig. 3d for different magnitudes of the SOC parameter k_h. Notably, the bands h_k indeed cross zero at $k_\mathrm{F} \approx k_{\mathrm{F},0}$, i.e. where the pairing is zero. This explains why the topological gap in Supplementary Fig. 3b is small, albeit the pairing amplitude in Supplementary Fig. 3c reaches values of up to 1 meV. One can derive analytically[7] that $k_\mathrm{F} = k_{\mathrm{F},0}$ for $A = 1$, $B = 0$ (i.e. for hybridizing YSR states, where an isolated state would be at exactly zero energy) and $k_{\mathrm{F},0} = 0.5\,\pi/d$. Thus, no band gap will open in this case (see also Fig. 1e of the main text). Since we have strong evidence that $k_{\mathrm{F},0} \approx 0.5\,\pi/d$, it is reasonable that the system presented in the main text is close to the gapless condition $k_\mathrm{F} = k_{\mathrm{F},0}$. This could explain the small magnitude of the *p*-wave pairing in our chains although there has been evidence that the Rashba SOC strength in Nb is significant[4] and an additional, effective Rashba term can be introduced by the inevitable inversion-symmetry breaking at the surface where the chain is located (see Supplementary Note 5). By this, an additional constraint is introduced for realizing strongly protected, i.e. isolated MMs in future experiments.

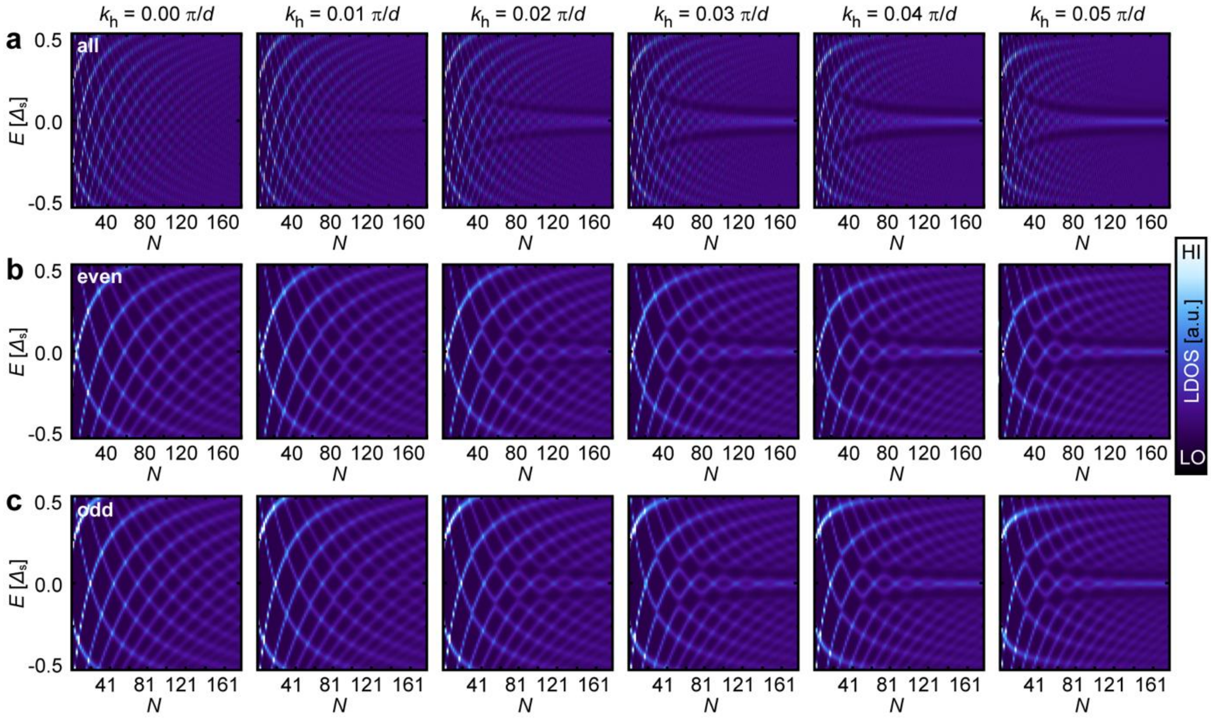

Supplementary Fig. 2 | Influence of the Rashba SOC strength k_h. **a**, LDOS on the first site of a finite chain of length *N* with increasing values of k_h as indicated above the panels. **b**, Dataset from **a** with only chains of even-*N* plotted in separate panels. **c**, Dataset from **a** with only chains of odd-*N* plotted in separate panels. For all simulations, the parameters from the main text and *T* = 200 mK were used.

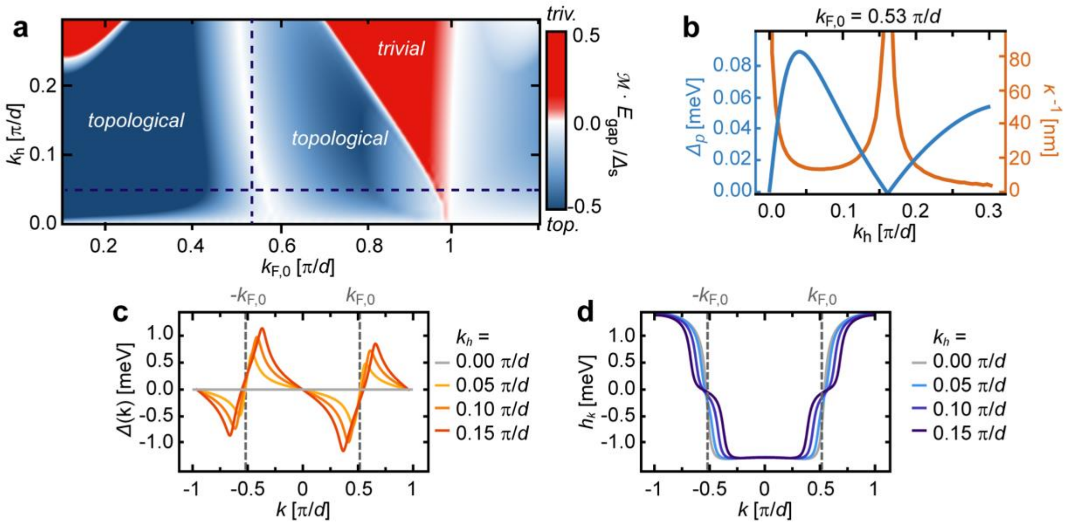

Supplementary Fig. 3 | Magnitude of the topological gap. **a**, Topological gap Δ_p of the model for different values of the effective Rashba SOC parameter k_h and the Fermi wavevector $k_{\mathrm{F},0}$. The dark dashed lines mark the parameters used in Fig. 4 of the main text to describe the experimental data. **b**, Topological gap Δ_p (blue) at the position of the vertical dashed line in panel **a** ($k_{\mathrm{F},0} = 0.53\,\pi/d$) for different values of the SOC parameter k_h and the decay length κ^{-1} (orange) determined by fitting the exponential long-range decay of the lowest energy wavefunction in a chain with *N* = 800 sites (see Eq. (S1)). **c**, *k*-dependence of the *p*-wave order parameter for different values of the SOC parameter k_h. **d**, Diagonal terms of the Hamiltonian h_k for different values of the SOC parameter k_h. For all simulations, the parameters from the main text were used.

Supplementary Note 4 | Overlap of the MMs with increasing chain length

As shown in Figs. 2b,c of the main text, localized zero energy states are found at the chain's ends for *N* = 32 whose phenomenology can be reproduced by the model for a single YSR band in Fig. 4b. These PMMs at

approximately zero energy presented in the main text feature several similarities with isolated MMs, such that they could be misinterpreted as strongly protected MMs: They have a good localization on the chain's ends, near-zero energy and are separated from higher energy excitations by a gap Δ_{FS}. The important difference is, that these states cannot be decomposed perfectly into spatially separated MMs localized on either chain ends[8], as we show in the following. We use the model presented in the Methods section and Supplementary Note 3 and transform the eigenvectors into the standard Majorana basis $\gamma_{L,j} = \frac{1}{\sqrt{2}}(c_j^\dagger + c_j), \gamma_{R,j} = \frac{1}{i\sqrt{2}}(c_j^\dagger - c_j)$. Here, $c_j^\dagger$ and c_j are the creation and annihilation operators of (Dirac-) fermionic states at site *j* of the chain. The result is shown in Supplementary Fig. 4 for short and long chains.

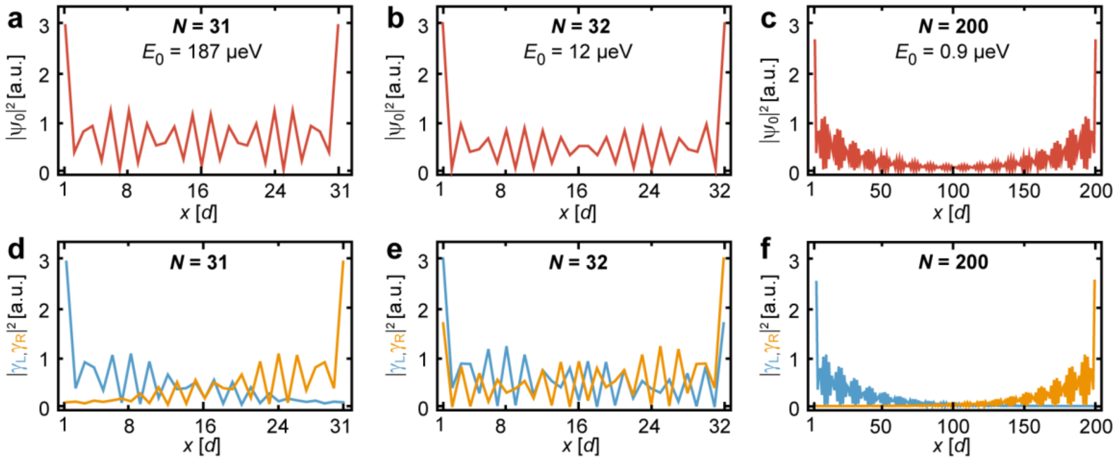

Supplementary Fig. 4 | Near-zero energy eigenstates in a Majorana basis. a-c, Absolute square of the lowest-energy (E_0) wave functions along finite chains of 31, 32 and 200 sites. **d-e**, Separation of the low energy states from panel **a-c** into Majorana operators γ_L, γ_R.

The Majorana decomposition of the near-zero energy mode in Supplementary Fig. 4b yields two strongly delocalized MMs with significant overlap (Supplementary Fig. 4e). Both wave functions are peaked on both ends, showing that the apparent localization of the features in Supplementary Fig. 4b is not a result solely from the topological nature of the end state. This is the reason for their weak protection against changes in the chain length. In contrast, the mode for *N* = 200 in Supplementary Fig. 4c can be separated into two well isolated MMs on the respective chain ends (Supplementary Fig. 4f). Interestingly, the separation (Supplementary Fig. 4d) for the finite-energy state in Supplementary Fig. 4a is better than that for the near-zero-energy state in Supplementary Fig. 4b. Although counterintuitive, the oscillating Majorana overlap and the energy of the Dirac state have been shown to anticorrelate in one-dimensional topological superconductors in the short wire limit[9,10].

Supplementary Note 5 | Majorana localization by inversion symmetry breaking

The good localization of the PMMs despite their strong, chain-length-dependent energy splitting is counterintuitive. In order to understand this behavior, we use a tight-binding toy-model approximating the low-energy band structure of a one-dimensional ferromagnetic chain on two layers of a superconducting substrate, in addition to the effective one-band model mentioned above and in the main text. The calculations were performed using the *Kwant* code[11]. This model thus reproduces the correct geometry of the experimental setup, where the chain is located on the surface of the superconductor and not in the bulk. The Bogoliubov-de-Gennes Hamiltonian for the chain reads:

$$\mathcal{H}_{\mathrm{chain}} = \sum_i c_i^\dagger(-J_c\tau_0 \otimes \sigma_z - \mu_c\tau_z \otimes \sigma_0 + \Delta_c\tau_x \otimes \sigma_0)c_i + \sum_{<i,j>} c_i^\dagger(-t_c\tau_z \otimes \sigma_0 + \alpha_c\tau_z \otimes \sigma_y)c_j \qquad (S2)$$

using the basis $c_i = \left(c_{i\uparrow}, c_{i\downarrow}, c_{i\downarrow}^\dagger, -c_{i\uparrow}^\dagger\right)^{\mathrm{T}}$. Here, J_{c} is the magnetic interaction strength, t_{c} is the hopping parameter in the chain, Δ_{c} is the proximity-induced superconducting order parameter in the chain, α_{c} is the strength of the Rashba SOC and μ_{c} is the chemical potential in the chain. τ_i and σ_i are the Pauli matrices in particle-hole- and

spin-space, respectively, and $\otimes$ represents the Kronecker product. $< i,j >$ indicates the summation over nearest neighbor sites and $c_{i,\sigma}^{\dagger}$ and $c_{i,\sigma}$ are the creation and annihilation operators of (Dirac-) fermionic states with spin σ at site *i* of the chain.

Two layers of a cubic substrate lattice with 70 x 15 sites are additionally considered,

$$\mathcal{H}_{\text{substrate}} = \sum_i c_i^{\dagger}(-\mu_s\tau_z \otimes \sigma_0 + \Delta_s\tau_x \otimes \sigma_0)c_i + \sum_{<i,j>} c_i^{\dagger}(-t_s\tau_z \otimes \sigma_0)c_j, \quad (S3)$$

and hopping between the chain and the substrate is taken into account,

$$\mathcal{H}_{\text{hop}} = \sum_{<i_c,j_s>} c_i^{\dagger}(-t_{cs}\tau_z \otimes \sigma_0)c_j. \quad (S4)$$

Similar to above, t_s is the hopping parameter in the substrate, Δ_s is the superconducting order parameter in the substrate, μ_s is the chemical potential in the substrate and t_{cs} is the hopping parameter between chain and substrate.

We compute the energy-dependent LDOS at site *x* by diagonalizing the Hamiltonian $\mathcal{H} = \mathcal{H}_{\text{chain}} + \mathcal{H}_{\text{substrate}} + \mathcal{H}_{\text{hop}}$ and summing over all pairs of eigenvalues E_i and eigenvectors ψ_i:

$$\text{LDOS}(E,x) \approx \sum_i |\psi_i(x)|^2 \left(-\frac{\partial f(E - E_i, T = 320 \text{ mK})}{\partial E}\right) \quad (S5)$$

with the Fermi-Dirac function $f(E,T)$ simulating the experimental thermal broadening. The summation over both the particle- and hole-components of the solutions in this section is justified by the value of $P(A,B) \approx 0.5$ for the δ-YSR states studied in the main text.

We start by reproducing the even-odd modulation of the sub-gap states using the following parameters, which result in a band structure with $k_F \approx 0.5\,\pi/d$ for $\alpha_c = 0$:

J_c = 0.7205 t_s, t_c = 0.55 t_s, μ_c = -0.55 t_s, Δ_c = 0.0, μ_s = -2 t_s, Δ_s = 0.13 t_s, t_{cs} = 0.7 t_s.

Calculating the topological invariant of the system[12,13] for these parameters, we find that it is non-trivial for all non-zero α_c. Adding an explicit Rashba SOC term $\alpha_c = 0.055\, t_s = 0.42\, \Delta_s$ in the chain, the band structure becomes gapped (see Supplementary Fig. 5a). This corresponds to a Rashba SOC parameter of $\hbar\lambda = 2d\alpha_c = 0.0059\ eV \cdot \text{Å}$, in good agreement with the model used in the main text. As shown in Supplementary Fig. 5b, we find localized PMMs in a chain of *N* = 32. However, similar to the model for YSR bands used in the main text and Supplementary Note 3, these PMMs split strongly in energy for chains with $N \leq 45$, as shown in Supplementary Figs. 5c,d. This splitting is modulated with a period of $\Delta N \approx 2$, yielding the same even-odd phenomenology as in the experiment.

In addition, we compare the localization of the edge modes for different chain-substrate couplings. If we set t_{cs} to zero, the chain is effectively one-dimensional and the substrate does not contribute to the LDOS on the chain. We quantify the localization of the low-energy modes by the ratio of the LDOS on the first site and the averaged LDOS in the chain's bulk,

$$\zeta = \frac{\text{LDOS}(E = 0, x = 1)}{2\,\overline{\text{LDOS}}(E = 0, 8 < x < 25)}, \quad (S6)$$

of a chain with *N* = 32. The LDOS is symmetric, hence it is equivalent to use the LDOS at the last site of the chain instead of the first site in the numerator of equation (S6). In Supplementary Figs. 6a,b, we compare the wavefunctions of the low-energy modes for the effective 1D model ($t_{cs} = 0$) and the extended model using a two-layer substrate (t_{cs} = 0.7 t_s). We find that the localization ζ for chains coupled to a substrate is consistently better than the localization in the purely one-dimensional model (see Supplementary Fig. 6c). Similar findings were reported in a model for Fe chains on Pb[13]. Surprisingly, when including the substrate, the lowest energy

modes are even localized for a vanishing explicit Rashba SOC term $\alpha_c = 0$. This is explained by the broken inversion-symmetry at the surface[14].

In the model used in the main text and Supplementary Note 3, the localization of the observed features can be explained by effective long-range hopping and pairing terms in the Hamiltonian, which are a result of an integration over the three-dimensional bulk of the superconducting host. Hence, the short-range decay of the MM wavefunction cannot be described as an exponential decay but is more complex[7] (see Supplementary Notes 3,4). Interestingly, in the limit of small coherence lengths ξ and weak Rashba SOC, all interactions are local and the LDOS corresponding to the low-energy states only slightly deviate from plane waves. Here, the YSR-band model converges to the one-dimensional case that is shown in Supplementary Fig. 6a.

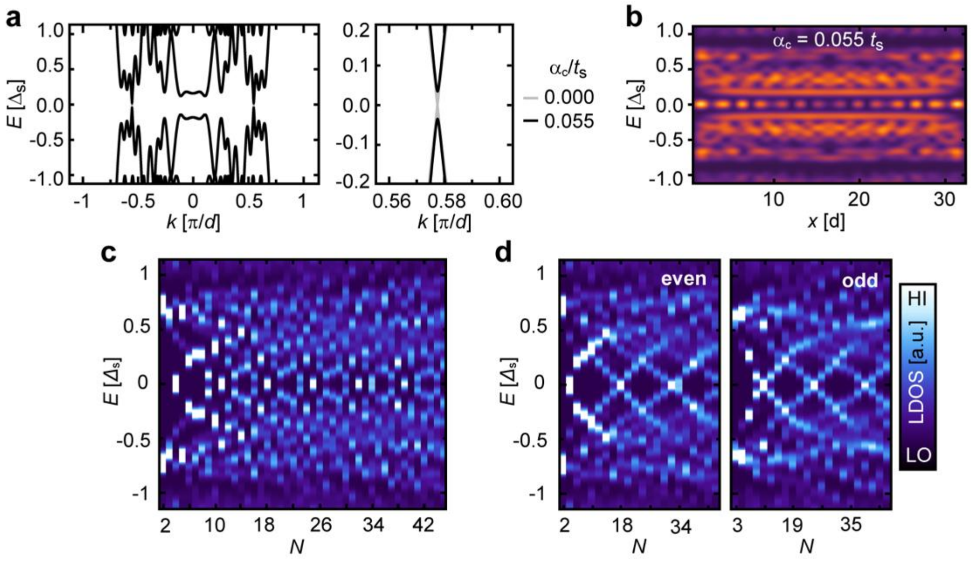

Supplementary Fig. 5 | Even-odd modulation in a magnetic chain on a substrate. a, Band structure of an infinite chain on a substrate, that is infinite in the direction of the chain, with and without SOC α_c in the chain, as indicated on the right. t_s and Δ_s are electron hopping and the superconducting order parameter in the substrate, respectively. The right panel shows the band structure around one of the Fermi points. **b**, LDOS along a chain of 32 sites using the parameters from **a** and $\alpha_c = 0.055 t_s$. **c**, LDOS on the first chain site in Mn_N chains of different number of sites N using the parameters from **b**. **d**, Dataset from **c** with chains of odd- and even-N plotted in separate panels.

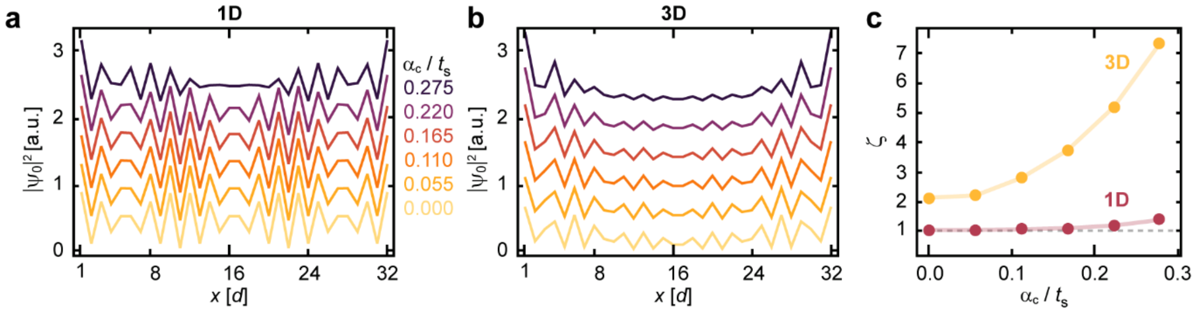

Supplementary Fig. 6 | Improved MM localization by inversion-symmetry breaking. a, Zero-energy LDOS without coupling to the substrate for different values of α_c. The curves are vertically offset for clarity. **b**, Same as in **a** but with finite coupling to two layers of a superconducting substrate. **c**, Localization ζ of the lowest-energy modes in the model without coupling to the substrate (1D) and the model with coupling to the substrate (3D), defined via the ratio of LDOS on the last site compared to the LDOS on the central sites, cf. Eq. (S6). $\zeta = 1$ corresponds to an entirely delocalized plane wave, as marked by the gray dashed line. Parameters: J_c = 0.7095 t_s, t_c = 0.55 t_s, μ_c = -0.55 t_s, Δ_c = 0.055 t_s, μ_s = -2 t_s, Δ_s = 0.13 t_s, t_{cs} = 0.7 t_s.

Supplementary Note 6 | Estimating the Fermi wavevector $k_{F,0}$

One input parameter for the model described in the Methods section of the main text and Supplementary Note 3 is the Fermi wavevector $k_{\mathrm{F},0}$ of the substrate hosting the YSR atoms. We can try to estimate the value of $k_{\mathrm{F},0}$ by probing the wavefunction of the single atom YSR state. The wavefunction has been calculated to oscillate far away from the impurity with $\psi(r) \propto \sin(k_{\mathrm{F},0}\, r)$ [15,16]. Since the measured LDOS is proportional to the absolute squares of the wavefunction, it is expected to be modulated with $q = 2\, k_{\mathrm{F},0}$. In Extended Data Fig. 3a, we show an example of a dI/dV map around a single Mn atom acquired at the energy of the δ_+-YSR state[4]. We computed the Fourier transform (FFT) of the map in Extended Data Fig. 3a with the central part removed to avoid effects from the orbital structure of the YSR state. The result is shown in Extended Data Fig. 3b and exhibits peaks at $q \approx (1.2 \pm 0.2)\,\pi/d$ and thus $k_{\mathrm{F},0} \approx (0.6 \pm 0.1)\,\pi/d$, compatible with $\pm\pi/2d$ as proposed by our results on Mn chains in the main text.

Supplementary Note 7 | Calculation of the YSR band structure within a single-band model based on first-principles simulations

We also determine the band structure of the YSR states using the model described in Ref. 4, which previously has been applied to Mn adatoms and dimers on Nb(110). The Hamiltonian of the system reads

$$H(k_\parallel) = H_\mathrm{b}(k_\parallel) + H_\mathrm{R}(k_\parallel) + H_\mathrm{sc}(k_\parallel) + H_\mathrm{imp}(k_\parallel), \qquad (S7)$$

where $k_\parallel$ denotes the wave number inside the one-dimensional Brillouin zone of the chain oriented along the $[1\bar{1}0]$ direction. The three-dimensional wave vector in the reciprocal space of bulk Nb is given by $\boldsymbol{k} = (\boldsymbol{k}_\perp, k_\parallel + G_\parallel)$, where $\boldsymbol{k}_\perp$ is the perpendicular component of the wave vector and $G_\parallel$ is a reciprocal lattice vector of the chain. The individual terms in the Hamiltonian represent the spectrum of bulk Nb

$$H_\mathrm{b}(k_\parallel) = \sum_{\boldsymbol{k}_\perp, G_\parallel} \Phi_{\boldsymbol{k}}^\dagger \xi_{\boldsymbol{k}} \tau^z \Phi_{\boldsymbol{k}}, \qquad (S8)$$

nearest-neighbor Rashba-type SOC on the bcc(110) surface of Nb

$$H_\mathrm{R}(k_\parallel) = \sum_{\boldsymbol{k}_\perp, G_\parallel} \Phi_{\boldsymbol{k}}^\dagger \left[4t_\mathrm{R} \sin\left(\frac{k^x}{2}a\right)\cos\left(\frac{\sqrt{2}}{2}k^y a\right)\sigma_4^y - 4t_\mathrm{R}\sin\left(\frac{\sqrt{2}}{2}k^y a\right)\cos\left(\frac{k^x}{2}a\right)\sqrt{2}\sigma_4^x\right]\Phi_{\boldsymbol{k}}, \qquad (S9)$$

s-wave superconducting pairing

$$H_\mathrm{sc}(k_\parallel) = \sum_{\boldsymbol{k}_\perp, G_\parallel} \Phi_{\boldsymbol{k}}^\dagger \Delta \tau^x \Phi_{\boldsymbol{k}}, \qquad (S10)$$

and the impurity potential

$$H_\mathrm{imp}(k_\parallel = k'_\parallel) = \frac{1}{N} \sum_{\mu,\alpha,\boldsymbol{k}_\perp, G_\parallel, \boldsymbol{k}'_\perp, G'_\parallel} \Phi_{\boldsymbol{k}}^\dagger \Psi_\mu(\boldsymbol{k}) \left(K^\mu \tau^z - \frac{J^\mu S}{2} S_\mu^\alpha \sigma_4^\alpha \tau^z\right) \Psi_\mu^*(\boldsymbol{k}')\Phi_{\boldsymbol{k}'}. \qquad (S11)$$

Note that $k_\parallel$ is fixed in all terms and the summations are restricted to $\boldsymbol{k}_\perp$ and $G_\parallel$. In Eqs. (S7)-(S11), $\Phi_{\boldsymbol{k}} = 1/\sqrt{2}\left(c_{\boldsymbol{k}\uparrow}, c_{\boldsymbol{k}\downarrow}, c_{-\boldsymbol{k}\downarrow}^\dagger, c_{-\boldsymbol{k}\uparrow}^\dagger\right)$ is the Nambu spinor, with the electron annihilation operators $c_{\boldsymbol{k}\sigma}$. The matrices in Nambu space read

$$\sigma_4^\alpha = \begin{bmatrix} \sigma^\alpha & 0 \\ 0 & -\sigma^\alpha \end{bmatrix}, \tau^z = \begin{bmatrix} \sigma^0 & 0 \\ 0 & -\sigma^0 \end{bmatrix}, \tau^x = \begin{bmatrix} 0 & \sigma^0 \\ \sigma^0 & 0 \end{bmatrix} \qquad (S12)$$

where σ^α are the usual 2×2 Pauli matrices for α = *x, y, z* and σ^0 is the unit matrix. The bulk Nb spectrum $\xi_{\boldsymbol{k}}$, the Rashba parameter t_R and the scattering potential shapes $\Psi_\mu(\boldsymbol{k})$ were determined based on *ab initio*

simulations in Ref. 4, where details about the procedure can be found. A finer mesh is used in reciprocal space compared to Ref. 4 due to the restriction to fixed $k_{\parallel}$ values because of the infinite chain. The order parameter Δ = 1.5 meV is based on the experimental value Δ_{Nb}. In the impurity potential, S is the spin quantum number, $\left(S_{\mu}^{x}, S_{\mu}^{y}, S_{\mu}^{z}\right)$ is the direction of the localized magnetic moment, and μ indexes the different scattering channels. The parameters K^{μ} and $J^{\mu}S/2$ are determined by an approximative procedure for the adatom discussed in Ref. 4, and the same values are used here for modelling the chain with and without SOC. The chain was assumed to be ferromagnetic along the z direction, and the calculations were restricted to the δ-YSR state.

The Green's function at complex energy z is expressed as

$$G(z,\boldsymbol{k},\boldsymbol{k}') = G_0(z,\boldsymbol{k})\delta_{\boldsymbol{k},\boldsymbol{k}'} + G_0(z,\boldsymbol{k})T(z,\boldsymbol{k},\boldsymbol{k}')G_0(z,\boldsymbol{k}). \qquad (S13)$$

Note that $G(z,\boldsymbol{k},\boldsymbol{k}')$ and $T(z,\boldsymbol{k},\boldsymbol{k}')$ are diagonal in the wave number of the chain ($k_{\parallel} = k'_{\parallel}$) due to the periodicity in real space, while the Green's function of the substrate

$$G_0(z,\boldsymbol{k}) = \left(z - \xi_{\boldsymbol{k}}\tau^z - 4t_{\mathrm{R}}\sin\left(\frac{k^x}{2}a\right)\cos\left(\frac{\sqrt{2}}{2}k^y a\right)\sigma_4^y + 4t_{\mathrm{R}}\sin\left(\frac{\sqrt{2}}{2}k^y a\right)\cos\left(\frac{k^x}{2}a\right)\sqrt{2}\sigma_4^x - \Delta\tau^x\right)^{-1} \qquad (S14)$$

is diagonal in $\boldsymbol{k}_{\perp}$ as well. The T operator is defined as

$$T(z,\boldsymbol{k},\boldsymbol{k}') = \sum_{\mu,\mu'} \Psi_{\mu}(\boldsymbol{k})\frac{1}{N}\left(K^{\mu}\tau^z - \frac{J^{\mu}S}{2}S_{\mu}^{\alpha}\sigma_4^{\alpha}\tau^z\right)\left[I_4 - \sum_{\boldsymbol{k}''_{\perp},G''_{\parallel}} \Psi_{\mu}^{*}(\boldsymbol{k}'')G_0(z,\boldsymbol{k}'')\Psi_{\mu'}(\boldsymbol{k}'')\frac{1}{N}\left(K^{\mu'}\tau^z - \frac{J^{\mu'}S}{2}S_{\mu'}^{\alpha}\sigma_4^{\alpha}\tau^z\right)\right]^{-1} \Psi_{\mu'}^{*}(\boldsymbol{k}'), \quad (S15)$$

where I_4 is the unit matrix in Nambu space.

The Shiba bands were determined by finding the poles of the T operator, i.e. the real energy values $|z| < \Delta$ for which the determinant of the matrix in Eq. (S15) vanishes. The results are summarized in Supplementary Fig. 7. Note that due to the particle-hole symmetry of the Hamiltonian, the YSR states always appear in pairs at positive and negative energies, and solid lines connect only the positive-energy or the negative-energy solutions as a guide to the eye. The minimal distance in energy between positive- and negative-energy states $2E_{\mathrm{gap}}$ may be interpreted as an upper bound for the induced gap in the system, limited by the resolution in $k_{\parallel}$. We found E_{gap} = 3 µeV in the absence (Supplementary Fig. 7a) and E_{gap} = 53 µeV in the presence of SOC (Supplementary Fig. 7b), respectively. The inner gap is more than a factor of ten higher when SOC is taken into account, and the spectrum is also flatter close to the minima than without SOC. This indicates that in the absence of SOC, the gap will close as the resolution in $k_{\parallel}$ is increased, but SOC may open an induced gap in the system. The magnitude of the gap here, which is based on first-principles calculations of the Rashba parameter, is comparable to the one obtained from the effective model in the main text (see also Supplementary Note 3).

The particle-hole symmetry makes it difficult to directly deduce the presence of band inversions in the Green's function method. To obtain further information, the local density of states (LDOS)

$$\mathrm{LDOS}(E,\mathrm{r},k_{\parallel}) = -\frac{1}{\pi}\mathrm{ImTr}\left[\frac{1}{N}\sum_{\boldsymbol{k}_{\perp},G_{\parallel},\boldsymbol{k}'_{\perp},G'_{\parallel}} e^{-\mathrm{i}(\boldsymbol{k}-\boldsymbol{k}')\mathrm{r}}\, G(E+\mathrm{i}\delta,\boldsymbol{k},\boldsymbol{k}')\frac{I_4+\tau^z}{2}\right] \qquad (S16)$$

and the magnetic density of states (MDOS)

$$\mathrm{MDOS}(E,\mathrm{r},k_{\parallel}) = -\frac{1}{\pi}\mathrm{ImTr}\left[\frac{1}{N}\sum_{\boldsymbol{k}_{\perp},G_{\parallel},\boldsymbol{k}'_{\perp},G'_{\parallel}} e^{-\mathrm{i}(\boldsymbol{k}-\boldsymbol{k}')\mathrm{r}}\, G(E+\mathrm{i}\delta,\boldsymbol{k},\boldsymbol{k}')\frac{I_4+\tau^z}{2}\sigma_4^z\right] \qquad (S17)$$

were calculated on the positions of the atoms in the chain, for each state indexed by $k_{\parallel}$ at the YSR energies E determined above. In Eqs. (S16) and (S17), δ denotes a small imaginary part, which was set to $k_{\mathrm{B}} \cdot 300\ \mathrm{mK}$ in order to approximate the experimentally observed energy resolution. The spin polarization SP of the states is

defined as the ratio of the MDOS and the LDOS. In the absence of SOC, the YSR states are expected to be fully spin-polarized as $\delta \rightarrow 0$, with opposite spin polarizations of the particle and hole states. Therefore, a sign change of the spin polarization when looking only at the positive energy states indicates a YSR band crossing the Fermi level. In the presence of SOC, the particle and hole states should hybridize, but far from the avoided crossings the sign of the spin polarization may still be used as an indication for whether a band crossing has occurred. This is illustrated by the color-coding in Supplementary Fig. 7. With SOC, the spin polarization changes sign at around $k_{\parallel} = 0.35\pi/d$, exactly at the position of the avoided crossing in Supplementary Fig. 7b. This value is not very far from the value $k_{\parallel,\mathrm{F}} = 0.53\pi/d$ used in the low-energy model calculations in Fig. 4 of the main text, taking into account that no fitting parameters were used for the chain calculations described here. Although the positive- and negative-energy bands are the closest for $k_{\parallel} = 0.97\pi/d$ in Supplementary Fig. 7b, there is no sign of an inversion of the spin polarization in this regime. In summary, the calculations indicate that the YSR bands cross the Fermi level only once for $k_{\parallel} > 0$, and this crossing is gapped out by the SOC. This supports the interpretation of the chain being in the topologically non-trivial regime.

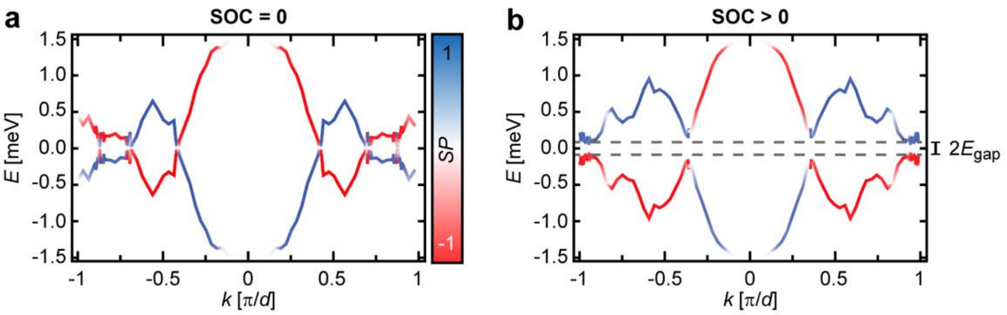

Supplementary Fig. 7 | Calculated YSR band structure in the chain from a first-principles based model. Band structure of the YSR states **a** without and **b** with SOC, using the model based on *ab-initio* calculations from Ref. 4. The spin polarization SP of the states is visualized by the color of the band, indicating a single band crossing in panel b for finite SOC.

Supplementary Note 8 | Interacting chains

Since we are constructing the chains by single atom manipulation, it is possible to create more complex nanostructures. As an example, we construct a junction of two Mn_N chains and control their interaction by the number of unoccupied sites $N_\emptyset$ between them (Fig. 5 of the main text). The full results for $0 \leq N_\emptyset \leq 5$ are shown in Extended Data Fig. 4. At large distances ($N_\emptyset > 5$), the chains do not interact strongly. Notice that the d*I*/d*V* line-profiles for the two respective chains with $N_\emptyset > 5$ are extremely similar, underlining the excellent reproducibility of our results. Moving the chains closer together results in small energy changes of the end state. Most notably, the energies of the left and right end states of the same respective chain change equivalently and symmetrically. This proves that it is a collective mode of the chain instead of a local electronic state. For $N_\emptyset$ = 1, we observe additional in-gap features between the chains which stem from hybridization between the states on the individual chains. This happens in particular at energies around $E \approx 800$ µeV and demonstrates that the interaction between the two chains is significant for these distances. Such additional in-gap states could hamper the identification of MMs in networks of chains if the experimental energy resolution is not sufficient. Finally, for $N_\emptyset$ = 0, the two chains merge into one Mn_{24} chain. Here, there is apparently no excessive d*I*/d*V* signal at the chain junction but only at the remaining two chain ends. However, the length of *N* = 24 accommodates PMMs with a finite energy splitting, as already discussed in detail in the main manuscript. Interestingly, while the higher-energy states appear to be spatially inhomogeneous (see $E \approx 600 - 800$ µeV), which we attribute to local variations in the substrate's chemical potential, the lowest energy mode remains symmetric in space, further indicating that it is a single quantum state. All these observed effects are in agreement with the expectations for PMMs.

Supplementary Note 9 | Deconvolution of d*I*/d*V* spectra taken with superconducting tips

In all experiments presented in the main text and the Supplementary Information, W tips covered with clusters of superconducting Nb were used (see Methods). While this is known to increase the experimental energy resolution, the d*I*/d*V* spectra measured with superconducting tips are not, as usually valid for metallic tips,

proportional to the sample's energy dependent LDOS $\rho_{\mathrm{s}}(E)$ at the tip apex position but to a convolution of $\rho_{\mathrm{s}}(E)$ with the tip DOS $\rho_{\mathrm{t}}(E)$:

$$\frac{\mathrm{d}I}{\mathrm{d}V}(V,T) \propto \int_{-\infty}^{\infty} \rho_{\mathrm{s}}(E)\frac{\partial\rho_{\mathrm{t}}(E+eV)}{\partial V}[f(E+eV,T)-f(E,T)]\mathrm{d}E$$

$$+\int_{-\infty}^{\infty} \rho_{\mathrm{s}}(E)\rho_{\mathrm{t}}(E+eV)\frac{\partial f(E+eV,T)}{\partial V}\mathrm{d}E \qquad (S18)$$

with the bias voltage *V* and the Fermi-Dirac distribution $f(E,T)$, dependent on both energy and the experimental temperature *T*. In the data shown in the main text and the Supplementary Information, we have used a numerical deconvolution procedure (see Ref. 1 for details) in order to regain $\rho_{\mathrm{s}}(E)$. This is done by dividing the integral in equation (S17) into 751 points in the energy interval $eV \in$ [-6 meV, +6 meV]. The measured spectrum d*I*/d*V*(*V*) is treated as a column vector $[\mathrm{d}I/\mathrm{d}V]^{\mathrm{SIS}}$ of length 751. Then, equation (S18) is equivalent to a matrix multiplication with the 751 × 751 matrix ***A***, defined by:

$$A_{i,j} = \frac{\partial\rho_{\mathrm{t}}(E_j+eV_i)}{\partial V}\left[f(E_j+eV_i,T)-f(E_j,T)\right]+\rho_{\mathrm{t}}(E_j+eV_i)\frac{\partial f(E_j+eV_i,T)}{\partial V}. \qquad (S19)$$

We compute the inverse $\boldsymbol{A}^{-1}$ of ***A*** and multiply the result with the vector $[\mathrm{d}I/\mathrm{d}V]^{\mathrm{SIS}}$, which approximately yields $\rho_{\mathrm{s}}(E)$. In the deconvolution process, the value *T* = 320 mK is used, as read out by a temperature sensor on the ^{3}He pot, which is thermally well coupled to the STM unit[17] (see Methods). In addition, the deconvolution can only be performed if $\rho_{\mathrm{t}}(E)$ is known.

Here, a broadened Dynes density of states with Δ_{t} = 1.42 meV is assumed for the superconducting Nb cluster on the tip:

$$\rho_{\mathrm{t}}(E) = \rho_0 Re\left[\frac{E-\mathrm{i}\Gamma}{\sqrt{(E-\mathrm{i}\Gamma)^2-{\Delta_{\mathrm{t}}}^2}}\right]. \qquad (S20)$$

ρ_0 is the normal conducting DOS, *Re* is the real part, and Γ is the Dynes broadening parameter. Γ = 0.01 meV was chosen for all tips used throughout this work, as validated by the observation of negative d*I*/d*V* features (see Supplementary Fig. 8), which are known to appear in the differential conductance when tunneling between very sharp features in $\rho_{\mathrm{s}}(E)$ and $\rho_{\mathrm{t}}(E)$. The values for Δ_{t} and Γ do not change drastically when the tip apex is modified (e.g. by indenting the tip several nm into the Nb surface), thereby suggesting that the Nb coating of the tip is stable and thick.

The value of Δ_{t} can be extracted from measurements of multiple Andreev reflections (MARs), which appear as very weak features in d*I*/d*V* spectra already at comparably large tip-sample distances. This can be seen in Supplementary Fig. 8: At $eV = \pm(\Delta_{\mathrm{s}}+\Delta_{\mathrm{t}}) = \pm 2.87\ \mathrm{meV}$, the convolution of the sample's and tip's coherence peaks yields a large peak in d*I*/d*V*. Zooming into the central gap region, we find additional peaks at $\pm 1.51\ \mathrm{mV}$ and $\pm 1.36\ \mathrm{mV}$, representing Andreev tunneling occurring at $eV = \pm\Delta_{\mathrm{s}}$ and $eV = \pm\Delta_{\mathrm{t}}$ for this particular tip[18]. Since the peak at $1.36\ \mathrm{mV}$ is observed to slightly change in energy for different microtips, we identify it with Δ_{t} and the peak at $1.51\ \mathrm{mV}$ with Δ_{s}, which is in good agreement with the bulk gap of Nb. For the tip used in the main text and the Supplementary Information, $\pm(\Delta_{\mathrm{s}}+\Delta_{\mathrm{t}}) = \pm 2.93$ meV and thus Δ_{t} = 1.42 meV is found. This value was used for equation (S20) and the numerical deconvolution process. Note that the MAR peaks are about three orders of magnitude smaller than the peaks at $\pm(\Delta_{\mathrm{s}}+\Delta_{\mathrm{t}})$, indicating that single-particle tunneling is the dominant transport channel. Therefore, MARs do not need to be considered in the deconvolution process.

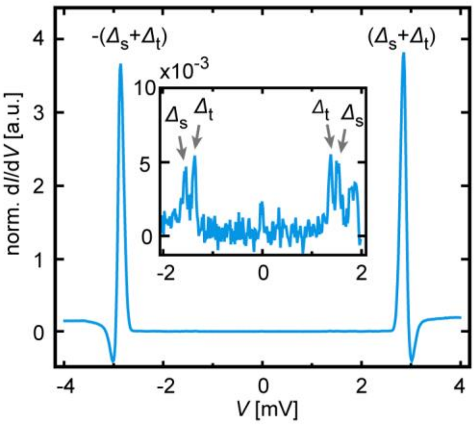

Supplementary Fig. 8 | Determination of the tip gap. dI/dV spectrum measured on the bare Nb(110) substrate (V = -6 mV, I = 1 nA, V_{mod} = 20 µV). In addition to the clear peaks at $\pm(\Delta_s + \Delta_t)$, additional features at $\pm\Delta_s$ and $\pm\Delta_t$ are found (inset).

References

1. Schneider, L. *et al.* Topological Shiba bands in artificial spin chains on superconductors. *Nat. Phys.* **17**, 943–948 (2021).
2. Nilius, N. Development of One-Dimensional Band Structure in Artificial Gold Chains. *Science* **297**, 1853–1856 (2002).
3. Fölsch, S., Hyldgaard, P., Koch, R. & Ploog, K. H. Quantum Confinement in Monatomic Cu Chains on Cu(111). *Phys. Rev. Lett.* **92**, 056803 (2004).
4. Beck, P. *et al.* Spin-orbit coupling induced splitting of Yu-Shiba-Rusinov states in antiferromagnetic dimers. *Nat. Commun.* **12**, 2040 (2021).
5. Schneider, L., Beck, P., Wiebe, J. & Wiesendanger, R. Atomic-scale spin-polarization maps using functionalized superconducting probes. *Sci. Adv.* **7**, eabd7302 (2021).
6. Peng, Y., Pientka, F., Glazman, L. I. & von Oppen, F. Strong Localization of Majorana End States in Chains of Magnetic Adatoms. *Phys. Rev. Lett.* **114**, 106801 (2015).
7. Pientka, F., Glazman, L. I. & von Oppen, F. Topological superconducting phase in helical Shiba chains. *Phys. Rev. B* **88**, 155420 (2013).
8. Stanescu, T. D., Lutchyn, R. M. & Das Sarma, S. Dimensional crossover in spin-orbit-coupled semiconductor nanowires with induced superconducting pairing. *Phys. Rev. B* **87**, 094518 (2013).
9. Escribano, S. D., Yeyati, A. L. & Prada, E. Interaction-induced zero-energy pinning and quantum dot formation in Majorana nanowires. *Beilstein J. Nanotechnol.* **9**, 2171–2180 (2018).
10. Peñaranda, F., Aguado, R., San-Jose, P. & Prada, E. Quantifying wave-function overlaps in inhomogeneous Majorana nanowires. *Phys. Rev. B* **98**, 235406 (2018).
11. Groth, C. W., Wimmer, M., Akhmerov, A. R. & Waintal, X. Kwant: a software package for quantum transport. *New J. Phys.* **16**, 063065 (2014).
12. Kitaev, A. Y. Unpaired Majorana fermions in quantum wires. *Physics-Uspekhi* **44**, 131–136 (2001).
13. Li, J. *et al.* Topological superconductivity induced by ferromagnetic metal chains. *Phys. Rev. B* **90**, 235433 (2014).
14. Bychkov, Y. A. & Rashba, E. I. Oscillatory effects and the magnetic susceptibility of carriers in inversion layers. *J. Phys. C Solid State Phys.* **17**, 6039–6045 (1984).
15. Rusinov, A. I. Superconductivity near a Paramagnetic Impurity. *ZhETF Pisma Redaktsiiu* **9**, 146 (1968).
16. Heinrich, B. W., Pascual, J. I. & Franke, K. J. Single magnetic adsorbates on s -wave superconductors. *Prog. Surf. Sci.* **93**, 1–19 (2018).
17. Wiebe, J. *et al.* A 300 mK ultra-high vacuum scanning tunneling microscope for spin-resolved spectroscopy at high energy resolution. *Rev. Sci. Instrum.* **75**, 4871–4879 (2004).
18. Ternes, M. *et al.* Subgap structure in asymmetric superconducting tunnel junctions. *Phys. Rev. B* **74**, 132501 (2006).
